# Photoacoustic Imaging for Women’s Gynecological Health: Advances and Clinical Prospects

**DOI:** 10.3390/bioengineering13040476

**Published:** 2026-04-18

**Authors:** Panangattukara Prabhakaran Praveen Kumar, Dong-Kwon Lim, Taeho Kim

**Affiliations:** 1 Department of Biomedical Engineering, Institute for Quantitative Health Science and Engineering, Michigan State University, East Lansing, MI 48824, USA; 2 KU-KIST Graduate School of Converging Science and Technology, Korea University, 145 Anam-ro, Seongbuk-gu, Seoul 02841, Republic of Korea; 3 KIST Brain Research Institute, Korea Institute of Science and Technology, 5 Hwarang-ro 14-gil, Seongbuk-gu, Seoul 02792, Republic of Korea

**Keywords:** photoacoustic imaging, contrast agents, women’s health, gynecological disorders, endometriosis, uterine disease, biomedical imaging, non-invasive diagnostics

## Abstract

Photoacoustic imaging (PAI) is an emerging hybrid biomedical imaging modality that combines the high molecular contrast of optical excitation with the deep tissue penetration of ultrasound detection. This review presents recent advances in PAI-based techniques for the detection and characterization of gynecological diseases in women, with particular focus on endometriosis and uterine-related disorders. We summarize the application of PAI across preclinical and translational studies, highlighting progress in photoacoustic microscopy, spectroscopic photoacoustic imaging, and endoscopic and probe-based implementations for non-invasive, high-resolution tissue evaluation. The role of functional and contrast-enhanced PAI approaches is discussed, emphasizing their ability to enhance diagnostic sensitivity, enable longitudinal monitoring, and provide detailed information on vascular, biochemical, and structural tissue characteristics. Furthermore, the expanding applications of PAI in assessing uterine, cervical, and ovarian pathologies, including tumor detection and tissue remodeling, are reviewed. Finally, current challenges, limitations, and future directions toward clinical translation are addressed. Collectively, this review underscores the potential of photoacoustic imaging as a powerful, non-invasive platform for early diagnosis, disease monitoring, and improved management of women’s health conditions.

## 1. Introduction

Gynecological disorders represent a significant and often underrecognized health burden among women of reproductive age, largely due to their gradual onset, non-specific clinical symptoms, and frequent recurrence [1,2]. Conditions such as endometriosis [3,4,5], uterine abnormalities [6,7], adenomyosis, and ovarian pathologies [8,9] such as cysts and malignancies are particularly prevalent and clinically impactful. For instance, endometriosis affects approximately 10% of reproductive-age women and is associated with chronic pelvic pain and infertility, whereas uterine fibroids are one of the leading causes of abnormal uterine bleeding and hysterectomy worldwide. Adenomyosis, often underdiagnosed, contributes to dysmenorrhea and menorrhagia, significantly impairing quality of life. These conditions frequently progress silently over extended periods before becoming clinically apparent, at which point treatment options are often more invasive and less effective.

Delayed diagnosis not only increases disease severity but also adversely affects fertility, quality of life, and long-term reproductive health outcomes [10,11]. Importantly, many of these conditions involve early-stage functional and microenvironmental changes, including altered vascularization, hypoxia, inflammation, and aberrant tissue remodeling, which are not readily detectable using conventional diagnostic tools. Current diagnostic approaches rely primarily on conventional imaging modalities, including ultrasound and magnetic resonance imaging (MRI), often complemented by invasive procedures such as laparoscopy and biopsy for definitive confirmation [12,13]. While these methods provide valuable anatomical information, they have inherent limitations. Ultrasound is operator-dependent and may lack sensitivity for deep or diffuse lesions (e.g., endometriosis), whereas MRI, although more sensitive, remains costly and less accessible. More critically, invasive techniques such as laparoscopy, which is considered the gold standard for endometriosis pose risks including infection, surgical complications, and patient discomfort, and are not suitable for routine screening or longitudinal monitoring [14]. Consequently, many gynecological diseases are identified only after substantial progression, reducing opportunities for early intervention and personalized therapeutic strategies. These limitations underscore the growing need for non-invasive, real-time imaging modalities that can detect early-stage disease and enable repeated assessments without risk to the patient. Non-invasive approaches offer several key advantages, including reduced patient burden, improved compliance, suitability for longitudinal monitoring, and the potential for earlier intervention before irreversible tissue damage occurs [15].

In this context, photoacoustic imaging (PAI), also known as photoacoustic tomography (PAT), has emerged as a promising modality capable of bridging the gap between structural and functional imaging. By combining optical absorption contrast with ultrasonic spatial resolution, PAI enables visualization of vascular remodeling, tissue oxygenation, hemoglobin concentration, and molecular changes associated with disease progression, with penetration depth of up to 5–7 cm [16,17,18]. This capability enables detection of pathological alterations at stages where conventional imaging is insensitive. Furthermore, PAI offers a non-invasive platform for disease detection, treatment guidance, and longitudinal monitoring, making it particularly well-suited for managing chronic gynecological conditions that require repeated evaluation [19,20]. With ongoing advances in probe design, imaging instrumentation, and multimodal integration, PAI holds substantial potential to transform the clinical management of gynecological disorders by shifting the diagnostic paradigm toward early detection, precision intervention, and improved patient outcomes [21,22].

Despite the growing body of literature on PAI, most existing reviews primarily focus on general oncological applications, vascular imaging, or instrumentation advances, with limited emphasis on gynecological diseases. Moreover, the integration of functional, molecular, and activatable probe-based photoacoustic strategies in the context of women’s health remains insufficiently addressed. This review distinguishes itself by providing a disease-focused and application-driven perspective, specifically targeting gynecological disorders such as endometriosis, uterine fibroids, and ovarian pathologies. In addition, we emphasize recent developments in activatable probes, multimodal imaging platforms, and quantitative photoacoustic biomarkers, alongside a critical discussion of translational challenges and clinical applicability. By bridging these aspects, this work aims to provide a more comprehensive and generalized framework for advancing PAI in women’s health.

## 2. Literature Search Methodology

A comprehensive literature search was conducted to collect relevant studies on PAI for gynecological applications. The primary databases used included Scopus, Web of Science, SciFinder, Liner Ai Research, and PubMed, ensuring broad coverage of both engineering and biomedical research. The search was performed using combinations of the following keywords: “photoacoustic imaging”, “photoacoustic tomography”, “gynecological disorders”, “endometriosis”, “uterine fibroids”, “adenomyosis”, “ovarian cancer”, “molecular imaging”, “activatable probes”, and “theranostics”. Boolean operators (AND/OR) were applied to refine the search results. Most of the included studies were published within the last 5–10 years (approximately 2015–2025) to capture recent technological and translational advances, although selected earlier seminal works were also included where necessary to provide foundational context. Articles were screened based on relevance to (i) gynecological disease applications, (ii) functional or molecular photoacoustic imaging, and (iii) translational or clinical potential. Both experimental and review articles were considered, with priority given to studies demonstrating clear imaging performance, biological validation, or clinical relevance to gynecological aspects.

## 3. Basic Principle and Fundamentals of Photoacoustic Imaging

### 3.1. Basic Principle of Photoacoustic Signal Generation

**Figure 1:**
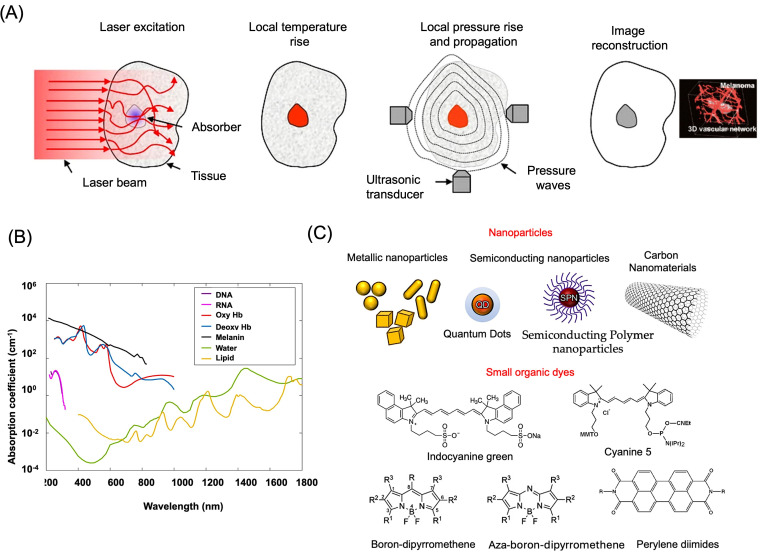
Basic principle for PAI. (**A**) Illustration of the fundamental working mechanism and image reconstruction process of PAI, along with a representative reconstructed photoacoustic image demonstrating melanoma visualization. Upon pulsed laser excitation, absorbed optical energy is converted into heat, resulting in a rapid local temperature rise. Under thermal and stress confinement, this induces thermoelastic expansion, generating an initial pressure rise that propagates as ultrasonic waves and is detected by transducers for image reconstruction. The red color in (**A**) represents the PA contrast agent. Reproduced with permission from [23]. Copyright 2016, Nature Publishing Group. (**B**) Absorption spectra of key endogenous chromophores present in biological tissues at physiologically relevant concentrations are shown. These include oxyhemoglobin (HbO_2_) and deoxyhemoglobin (HbR) at approximately 150 g/L in blood, lipids comprising about 20% tissue volume, and water representing nearly 80% of tissue composition. Additional absorbers include nucleic acids (DNA and RNA) at ~1 g/L within cell nuclei, melanin at roughly 14.3 g/L in average human skin, reduced and oxygenated myoglobin (MbR and MbO_2_) at approximately 0.5% by mass in skeletal muscle, and bilirubin at a concentration of about 12 mg/L in blood. Reproduced with permission from [24]. Copyright 2014, Elsevier. (**C**) Pictorial representation for the commonly used exogenous contrast agents for PAI.

PAI is a hybrid optical–acoustic imaging modality that enables high-contrast visualization of biological tissues by converting absorbed optical energy into ultrasonic signals. Upon nanosecond-pulsed laser-light irradiation, photoabsorbing molecules undergo rapid nonradiative relaxation, resulting in localized temperature increases that induce transient thermoelastic expansion and generate acoustic pressure waves [21]. These ultrasound signals propagate through tissue and are detected by ultrasonic transducers, allowing image reconstruction based on signal intensity and time-of-flight characteristics (Figure 1A) [23]. In contrast to conventional ultrasound imaging, which relies primarily on differences in mechanical impedance, PAI exploits variations in optical absorption, thereby providing enhanced contrast for imaging chromophores within complex biological environments. Compared with purely optical imaging techniques, PAI achieves substantially greater penetration depth extending to several centimeters while maintaining micrometer-scale spatial resolution. Photoacoustic contrast can originate from endogenous absorbers, such as hemoglobin and melanin, or from exogenously administered agents including organic dyes, fluorophores, and nanostructured materials (Figure 1B) [24,25]. However, endogenous chromophores are often present at limited or heterogeneous concentrations, which can restrict sensitivity and reproducibility. Consequently, exogenous contrast agents are frequently employed to amplify photoacoustic signals and improve imaging reliability (Figure 1C) [26].

The initial pressure rise (P_0_) generated during photoacoustic signal formation can be described by the following relationship:

(1) P_0_ = Γη_th_ μ_a_ F

where Γ reflects the efficiency of converting temperature rise into pressure, linking thermal and mechanical responses of tissue, η_th_ corresponds to the fraction of absorbed optical energy converted into heat, μ_a_ represents the optical absorption coefficient of the photoabsorber, and F is the local optical fluence. This expression highlights that photoacoustic signal intensity is determined by both the absorption efficiency and photothermal conversion capability of the contrast agent. Efficient photoacoustic signal generation requires that optical energy deposition occurs under thermal and stress confinement conditions, where the laser pulse duration is shorter than both the thermal diffusion time and stress relaxation time of the tissue. Under these conditions, the deposited energy remains spatially confined, resulting in a rapid temperature rise that is directly converted into an initial pressure increase without significant heat dissipation. This pressure rise generates broadband ultrasonic waves that propagate through tissue and are detected by ultrasound transducers.

Accordingly, the rational design of photoacoustic probes, particularly organic dyes and dye-based nanomaterials, that exhibit strong absorption and high photothermal efficiency, is critical for optimizing PAI performance. Beyond structural imaging, PAI effectively addresses key limitations of traditional optical techniques by minimizing photon scattering in biological tissues, enabling high-resolution imaging at deep tissue penetration [27,28,29]. A distinctive advantage of PAI is its ability to perform multi-wavelength spectroscopic imaging, which allows quantitative assessment of physiologically important parameters such as tissue oxygenation [30], vascular dynamics [31], and metabolic activity [32]. Furthermore, PAI can be readily integrated with other imaging modalities, including ultrasound and fluorescence imaging, enabling multimodal and multiscale imaging platforms that span spatial resolutions from the cellular level to whole organs [33,34]. Its rapid data-acquisition capability also facilitates real-time monitoring of dynamic biological processes, such as blood perfusion and drug distribution.

Owing to these advantages, PAI has emerged as a valuable tool in both preclinical and clinical biomedical research, with demonstrated applications in oncology [35], neurology [36], vascular biology [37], and inflammatory diseases [38]. In cancer imaging, PAI provides access to functional features of the tumor microenvironment that are difficult to capture using conventional imaging approaches alone. These include vascular morphology, perfusion patterns, and oxygenation heterogeneity, all of which are closely associated with tumor progression, therapeutic resistance, and disease outcome. By leveraging the distinct optical absorption spectra of oxygenated and deoxygenated hemoglobin, PAI enables non-invasive assessment of tissue hypoxia, a hallmark of aggressive and treatment-resistant tumors. Additionally, single-wavelength imaging strategies can be employed to visualize tumor-associated vasculature [39] and monitor angiogenic remodeling [40], while quantitative parameters such as vessel density [41], diameter [42], and geometric complexity serve as imaging-derived biomarkers for longitudinal disease evaluation [27].

### 3.2. Imaging Configurations

Depending on the imaging depth, resolution and application context, PAI can be implemented using multiple imaging configurations. These configurations broadly include photoacoustic microscopy, photoacoustic computed tomography, and endoscopic or probe-based photoacoustic systems, each offering distinct advantages for biological and biomedical investigations.

#### 3.2.1. Photoacoustic Microscopy (PAM)

Photoacoustic microscopy (PAM) represents a high-resolution implementation of PAI designed to visualize biological structures within shallow to moderate tissue depths [43]. In PAM, imaging is performed in optical regimes where light propagation is partially ballistic or weakly diffusive, enabling detailed visualization of micro-scale features. PAM is generally characterized by spatial resolutions finer than tens of micrometers, well below the resolving capability of the unaided human eye. A defining distinction between PAM and photoacoustic computed tomography lies in the image formation strategy: PAM typically employs a focused, single-element ultrasound detector to acquire spatially resolved signals, whereas tomographic approaches rely on multi-element detector arrays and computational reconstruction algorithms.

**Figure 2:**
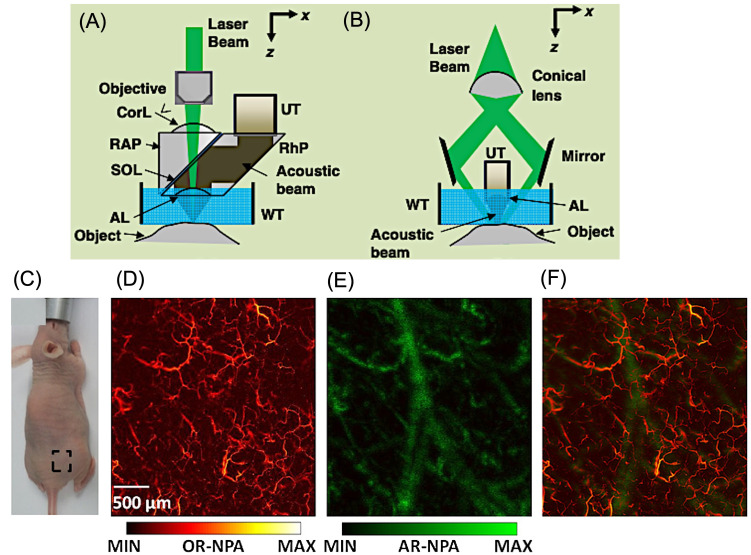
Optical Resolution Photoacoustic microscopy for in vivo imaging. (**A**) Schematic illustration of a second-generation optical-resolution photoacoustic microscopy (2G-OR-PAM) system, in which lateral spatial resolution is governed by diffraction-limited optical focusing. Components include an acoustic lens (AL), correction lens (CorL), right-angled prism (RAP), rhomboid prism (RhP), silicone oil layer (SOL), ultrasonic transducer (UT), and water tank (WT). (**B**) Configuration of dark-field acoustic-resolution photoacoustic microscopy (AR-PAM), where lateral resolution is primarily defined by diffraction-limited acoustic focusing. (**C**) Photograph of the mouse indicating the selected imaging area outlined by a dashed box. (**D**) Optical-resolution PAM (OR-PAM) image showing superficial skin vasculature. (**E**) Acoustic-resolution PAM (AR-PAM) image highlighting deeper vascular structures. (**F**) Merged PAM image combining both datasets. OR-NPA represents the normalized photoacoustic amplitude obtained from the OR-PAM image, while AR-NPA denotes the normalized photoacoustic amplitude from the AR-PAM image. Reproduced with permission from [48]. Copyright 2013, Optica Publishing Group.

The spatial resolution of PAM is governed by both optical excitation and acoustic detection parameters. Lateral resolution arises from the combined influence of the illumination profile, and the acoustic point spread function, while axial resolution is dictated primarily by the bandwidth of the ultrasound transducer. Based on the dominant contributor to lateral resolution, PAM systems are commonly categorized into optical-resolution PAM (OR-PAM) and acoustic-resolution PAM (AR-PAM) [43,44]. In OR-PAM, the excitation beam is tightly focused on a diffraction-limited spot that is significantly smaller than the acoustic focal zone, making the optical focus the primary determinant of lateral resolution. As a result, OR-PAM achieves exceptionally fine spatial resolution but is restricted to superficial imaging depths due to strong optical scattering in tissue (Figure 2A) [45,46]. Conversely, AR-PAM employs a broader optical illumination that encompasses the acoustic focal region, with lateral resolution primarily defined by ultrasonic focusing. This configuration allows imaging at deeper penetration with reduced sensitivity to optical scattering, at the cost of spatial resolution (Figure 2B) [47]. As illustrated in Figure 2C, an in vivo scan was performed on the dorsal region of an athymic nude mouse. Optical-resolution (OR) and acoustic-resolution (AR) PAM (Figure 2D,E) were collected simultaneously during a single scanning session, using step sizes of 2 μm and 20 μm and laser pulse repetition rates of 20 kHz and 2 kHz, respectively. The AR-PAM image captured prominent, deeper blood vessels but resolved fewer superficial microvessels compared with the OR-PAM image. In contrast, OR-PAM provided high-resolution visualization of small, shallow vasculature that was not clearly detected in AR mode. The combined image from Figure 2F, integrates both datasets, revealing superficial microvascular details from OR-PAM alongside deeper vascular structures from AR-PAM [48].

OR-PAM has also been explored as a potential “optical biopsy” tool, enabling high-resolution, label-free imaging of microvascular and tissue morphology at near-cellular resolution [49,50]. By providing real-time structural and functional information without the need for tissue excision, OR-PAM offers a promising non-invasive alternative to conventional biopsy for early disease detection and monitoring [51]. In addition to high-resolution structural imaging, PAM has demonstrated potential in guiding diagnostic decision-making in a manner analogous to optical biopsy. For example, PAM has been employed for sentinel lymph node (SLN) mapping, which is a critical step in cancer staging. Using contrast-enhanced approaches, studies have demonstrated the ability to non-invasively identify SLNs and lymphatic vessels at millimeter-scale depths, enabling precise localization of disease-relevant tissue without the need for invasive procedures [52,53]. Furthermore, targeted nanoparticle-based PAM imaging has enabled molecular-level detection and monitoring of tumor-associated biomarkers, supporting real-time evaluation of disease status and treatment response. Although these approaches are not direct substitutes for histopathological biopsy, they highlight the potential of PAM-based techniques as non-invasive tools for tissue characterization and image-guided diagnostics.

PAM architectures can be further adapted for internal organ imaging applications through miniaturization, leading to the development of photoacoustic endoscopy (PAE). These systems often utilize rotational or linear scanning mechanisms and can operate in either optical- or acoustic-resolution modes, depending on the design of the illumination and detection components. By systematically adjusting optical parameters such as numerical aperture and excitation wavelength, or acoustic parameters including transducer frequency and focusing geometry, PAM platforms can be tuned across a wide range of resolutions and depths. This scalability makes PAM a versatile tool for studying microvascular structures, tissue morphology, and functional biomarkers.

#### 3.2.2. Photoacoustic Computed Tomography (PACT)

In photoacoustic computed tomography (PACT), image formation relies on mechanical or electronic scanning of multi-element ultrasound detector arrays, followed by computational reconstruction of the acquired signals [44]. Unlike photoacoustic microscopy (PAM), where signals arise from a spatially confined focal volume and can be directly mapped along the acoustic axis, PACT detectors possess broad angular sensitivity and require data integration from multiple elements to reconstruct an image. Based on detector arrangement, PACT systems are commonly categorized into planar, cylindrical, and spherical geometries, each offering distinct imaging capabilities.

**Figure 3:**
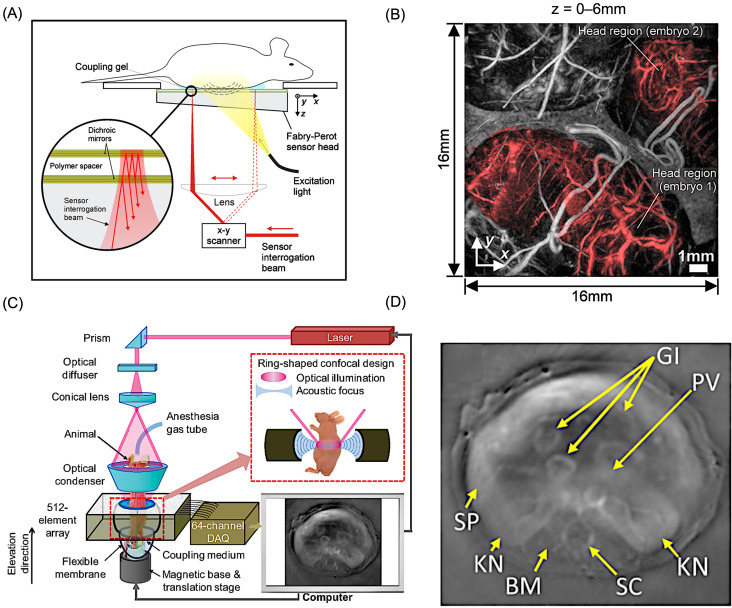
Photoacoustic Computed Tomography Imaging system for in vivo applications. (**A**) Schematic representation of a Fabry–Perot interferometer-based photoacoustic computed tomography (PACT) system. Photoacoustic signals are generated following absorption of nanosecond pulses from a wavelength-tunable optical parametric oscillator (OPO) laser and are detected using a transparent polymer Fabry–Perot ultrasound sensor. The sensor consists of two dichroic mirror layers separated by an approximately 40 µm polymer spacer, forming the interferometric cavity. Acoustic waves are recorded by raster-scanning a continuous-wave (CW) focused interrogation laser across the sensor surface, where acoustically induced changes in cavity reflectivity are measured at each position to generate two-dimensional maps. (**B**) Maximum-amplitude projection of the reconstructed three-dimensional dataset (0–6 mm depth), highlighting two embryos (shown in red). Reproduced with permission from [54]. Copyright 2012, SPIE Digital Library. (**C**) Experimental configuration of a full-ring confocal whole-body photoacoustic computed tomography (RC-PACT) system. A schematic overview of the imaging setup is presented, with the dashed inset illustrating a cross-sectional view of the confocal geometry. (**D**) Representative in vivo RC-PACT images of athymic mice obtained non-invasively in the kidney region. Labeled anatomical structures include backbone muscle (BM), gastrointestinal tract (GI), kidney (KN), liver (LV), portal vein (PV), spinal cord (SC), and spleen (SP). Reproduced with permission from [55]. Copyright 2012, SPIE Digital Library.

Planar-view PACT systems typically employ two-dimensional piezoelectric transducer arrays or optical sensors such as Fabry–Perot interferometers, enabling wide-field signal acquisition (Figure 3A) [54]. While optical interferometric detectors offer high sensitivity and fine spatial sampling, their sequential readout can limit imaging speed compared to array-based approaches. An example of in vivo abdominal imaging obtained using a Fabry–Perot interferometer-based PACT system is illustrated in Figure 3B. The image clearly delineates two developing embryos (red color), together with surrounding uterine blood vessels and overlying skin structures, demonstrating the capability of this approach to resolve both anatomical and vascular features in a pregnant mouse model [54]. Cylindrical-view PACT, often implemented using ring-shaped transducer arrays, provides efficient cross-sectional imaging and can be extended to three-dimensional visualization through axial scanning (Figure 3C) [55,56]. This configuration is particularly suitable for rapid two-dimensional imaging of dynamic physiological processes and for visualizing vascular structures within deep tissues. Figure 3D illustrates the configuration of a ring-array-based small-animal imaging platform along with representative reconstructed images [55]. Organs with high vascular content, including the liver, spleen, spine, kidneys, and gastrointestinal tract, are distinctly visualized. Fine vascular networks within these structures are also resolved, demonstrating the system’s capability for high-resolution angiographic imaging.

Spherical-view PACT systems employ transducer arrays arranged over curved geometries to achieve near-isotropic spatial resolution within the central imaging volume [57,58]. Although these systems can deliver high-quality volumetric images, they typically require mechanical scanning and full data acquisition prior to image reconstruction, which can limit real-time imaging performance. Recent advances in reconstruction algorithms have partially alleviated these limitations by enabling dynamic imaging from sparsely sampled datasets [27]. Collectively, the flexibility of PACT detection geometries allows the technique to be adapted for diverse preclinical and translational imaging applications.

#### 3.2.3. Endoscopic and Probe-Based PAI

Photoacoustic endoscopy (PAE) enables minimally invasive PAI by integrating optical excitation and acoustic detection within compact endoscopic probes [59,60]. This configuration allows localized visualization of tissue composition, vascular architecture, and functional biomarkers inside luminal organs, including the gastrointestinal and reproductive systems. When combined with ultrasound imaging, PAE provides complementary structural and functional information, supporting real-time guidance during endoscopic procedures. Early PAE platforms demonstrated intraluminal PAI but were limited by single-modality operation and slow acquisition speeds. Subsequent developments introduced dual- and multimodal probes incorporating photoacoustic, ultrasound, and fluorescence imaging, enabling three-dimensional visualization of vascular and luminal anatomy [61]. Continued probe miniaturization improved compatibility with standard endoscopic channels, advancing its translational potential [62].

Although PAE has been explored for many years as a clinical extension of PAI, several technical challenges have hindered its widespread clinical adoption [63]. Recent studies, however, demonstrate meaningful progress toward overcoming these barriers, particularly in probe design and light–acoustic integration. One of the primary challenges in multimodal PAE systems is achieving efficient, stable light delivery within the compact endoscopic geometries. To address this, Wen and co-workers developed a disposable photoacoustic–ultrasound (PAUS) endoscopic catheter coupled with a dedicated power interface, enabling switchable operation, internal three-dimensional scanning, and reproducible performance for gastrointestinal imaging [64]. By optimizing optical relay design, they significantly reduced waveguide insertion losses while maintaining high optical power delivery. Their focus-adjustable acousto-optic coaxial probe enabled high-sensitivity optical-resolution imaging, achieving real-time visualization of colonic microvasculature and tissue layering at micrometer-scale resolution in rats, highlighting its promise for the detection of gastrointestinal disease.

Complementary advances have focused on improving probe adaptability to the endoscopic environment. Zhu and colleagues introduced a hydrostatic balloon-based catheter incorporating a miniaturized ultrasound array and angled optical fiber, allowing conformal acoustic coupling along irregular intestinal surfaces [63]. This design enabled effective photoacoustic evaluation of inflammatory and fibrotic intestinal conditions and demonstrated sufficient depth for in vivo differentiation of normal and pathological tissue states. Notably, the collapsible balloon configuration is compatible with conventional ileocolonoscopy, underscoring the translational potential of such PAE systems for clinical gastrointestinal diagnostics.

The fundamental trade-offs between spatial resolution and depth have led to two principal PAE modes: acoustic-resolution systems optimized for deeper imaging and optical-resolution systems that achieve higher lateral resolution at superficial depths [65]. Recent advances in scanning strategies, autofocusing mechanisms, and panoramic imaging geometries have improved imaging speed, resolution, and robustness. More recently, optical-scanning-based and Fabry–Pérot-based probes have enabled compact, high-resolution PAE systems with enhanced signal-to-noise ratios, facilitating in vivo assessment of vascular dynamics and tissue oxygenation [66]. Collectively, these developments position PAE as a promising tool for functional, image-guided endoscopic diagnostics.

Given the diverse configurations of photoacoustic imaging systems, a comparative summary of their key parameters, including resolution, penetration depth, and application scope, is provided in Table 1 to highlight their respective strengths and limitations.

Among these modalities, PACT and probe-based systems show the greatest potential for clinical translation, particularly when combined with molecular and activatable contrast agents which are discussed in the next section.

## 4. Functional and Molecular Imaging Capability of PAI

Functional PAI exploits the intrinsic optical absorption of endogenous chromophores, including hemoglobin, lipids, and water, to non-invasively assess physiological and metabolic processes [26,67]. Multispectral acquisition allows quantitative mapping of parameters such as blood oxygen saturation, hemoglobin concentration, and hemodynamic changes, providing valuable insight into tissue perfusion, oxygen utilization, and organ function. In contrast, contrast-enhanced PAI employs exogenous photoabsorbing agents to further improve sensitivity and molecular specificity [68,69]. These agents, ranging from small organic dyes to engineered nanoparticles, are designed to generate strong photoacoustic signals, enabling visualization of weakly absorbing structures and targeted molecular events. Continued advances in contrast agent design are expanding the diagnostic and theranostic potential of PAI, supporting its growing role in biomedical imaging and precision medicine.

### 4.1. Endogenous Contrast Mechanisms

**Figure 4:**
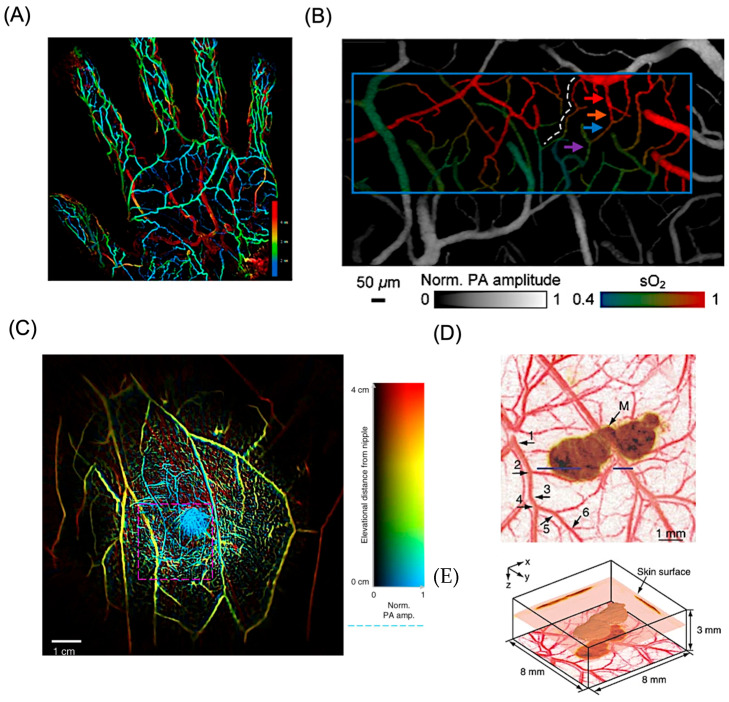
Endogenous contrast-based PAI application. (**A**) Maximum-intensity projection photoacoustic (PA) image showing the vascular network of the palm acquired at 795 nm, where the color scale indicates imaging depth. Reproduced with permission from [71]. Copyright 2018, Nature Publishing group. (**B**) Photoacoustic oxygen saturation map of the somatosensory cortex recorded during electrical stimulation. The blue box highlights the selected region of interest used for subsequent functional imaging analysis. Red, blue, and purple arrows indicate representative locations along the vascular pathway, corresponding to arteriole, capillary, and venule segments, respectively. Reproduced with permission from [83]. Copyright 2020, SPIE. (**C**) Photoacoustic computed tomography image illustrating the vascular architecture of the right breast in a healthy female volunteer, with depth information represented using a color-coded scale. Reproduced with permission from [84]. Copyright 2018, Nature Publishing group. (**D**) Composite maximum-amplitude projection (MAP) images obtained by projecting the maximum photoacoustic signals along the z-axis. Blood vessels (584 nm) are pseudo-colored red, and melanoma (764 nm) is pseudo-colored brown. Six hierarchical levels of vascular branching (1–6) are visible. (**E**) Three-dimensional reconstruction of the melanoma from 764 nm data. MAP images projected along the x-and y-axes are shown on the side walls, and the composite image from (**D**) is displayed at the bottom. The tumor surface is located 0.32 mm below the skin, with a thickness of 0.3 mm. Reproduced with permission from [94]. Copyright 2006, Nature Publishing group.

Several intrinsic tissue components, including hemoglobin, melanin, lipids, and water, serve as endogenous chromophores for photoacoustic imaging, enabling label-free visualization of physiological and structural features (Figure 4). Among these, hemoglobin is the dominant absorber in the visible and near-infrared spectral regions and plays a central role in functional photoacoustic imaging [70]. These endogenous chromophores exhibit distinct absorption profiles across the near-infrared (NIR) window (approximately 700–1100 nm), allowing wavelength selection to optimize photoacoustic contrast from specific tissue constituents. The distinct optical absorption spectra of oxygenated (HbO_2_) and deoxygenated hemoglobin (Hb) enable multispectral photoacoustic imaging to quantify blood oxygen saturation and hemoglobin concentration. This allows simultaneous mapping of vascular architecture [71,72] and tissue oxygenation [73,74], providing insight into hemodynamic function [75,76], perfusion [77,78], and hypoxia [79]. Such capabilities have been widely applied in studies of tumor hypoxia [79], ischemic injury [80,81], and tissue regeneration [73,82].

Representative demonstrations of blood-derived contrast in PAI are illustrated in Figure 4. One example shows a depth-resolved maximum-intensity projection of vascular networks in the human palm acquired at 795 nm, where color mapping indicates vessel depth (Figure 4A) [71]. This image reveals clear stratification of the vascular anatomy, with major arterial structures such as the superficial palmar arch and digital arteries positioned deeper within the tissue than the superficial venous network. In addition to structural visualization, PAI supports functional assessment of tissue physiology. As shown in Figure 4B, wavelength-dependent imaging enables mapping of blood oxygen saturation within the somatosensory cortex during electrically evoked neural activity, allowing simultaneous observation of microvascular architecture and dynamic oxygenation changes [83]. This ability to image both normal and pathological vasculature underlies the value of PAI in cancer detection, particularly in breast tissue. An example of whole-breast vascular imaging is presented in Figure 4C, where depth-encoded visualization provides three-dimensional insight into vascular distribution throughout the tissue, offering a comprehensive view of breast vasculature relevant for diagnostic evaluation [84].

The strong hemoglobin contrast also makes PAI exceptionally powerful for characterizing vascular structure and function across different spatial scales [85]. From resolving microvascular networks to assessing larger vessel architecture, PAI provides scalable resolution at depths beyond the reach of purely optical techniques. This capability has been widely applied to study vascular remodeling in tumors, where abnormal vessel organization and hypoxia are hallmarks of disease progression, as well as in metabolic disorders such as diabetes, where micro- and macrovascular dysfunction play a central role [35,42]. In addition, PAI has demonstrated promise in imaging specialized vascular beds, including lymph nodes and chorioretinal tissues, highlighting its potential for both basic biological discovery and future clinical translation in vascular diseases [86].

Melanin also produces intense photoacoustic signals and has been successfully explored for melanoma detection [87]. Melanin has a broad absorption spectrum that spans from visible light to near-infrared region (NIR) wavelengths (400–1064 nm) and thus allows for a wide range of excitation wavelengths to be used in PAI. Melanin present in ocular tissues such as the uvea and is highly concentrated in pigmented tumors, including ocular melanoma [88]. PAI studies have shown that melanin generates stronger signals at shorter wavelengths (e.g., 532 nm) compared to longer wavelengths (e.g., 1064 nm), reflecting its wavelength-dependent absorption, while longer wavelengths provide greater imaging depth [89]. High-resolution photoacoustic microscopy has further enabled differentiation between retinal pigment epithelium and choroidal melanin, aided by optical modeling approaches to optimize spatial resolution [90,91]. Beyond ocular applications, photoacoustic detection of melanin has been explored for identifying circulating melanoma cells, offering potential benefits for melanoma diagnosis and treatment monitoring [92]. While near-infrared wavelengths can enhance contrast between melanin and hemoglobin, limited penetration restricts detection of thicker or deeply located lesions. Spectral overlap between melanin and blood remains a challenge, though advanced spectral analysis and three-dimensional image reconstruction approaches have improved sensitivity and enabled visualization of small melanoma features that are difficult to resolve using conventional two-dimensional imaging. Langhout and co-workers advanced spectral photoacoustic analysis by systematically evaluating the absorption signatures of multiple chromophores across the 680–970 nm range [93]. While this approach enabled differentiation between hemoglobin and melanin, detection of small melanoma lesions remained challenging due to insufficient photoacoustic signal intensity, resulting in poor agreement with the expected spectral signature. This limitation was later addressed by Zhang and colleagues, who employed depth-resolved reconstruction of photoacoustic z-stacks acquired at 764 nm to generate three-dimensional morphological representations (Figure 4D) [94]. The resulting volumetric imaging strategy improved feature visibility and allowed identification of melanoma structures that were not discernible using conventional two-dimensional PAI (Figure 4E).

In contrast, although lipids, collagen, elastin, and water can contribute to endogenous photoacoustic contrast and are valuable in other biological contexts, their relevance in oncology remains limited [89]. Their comparatively weaker absorption in the near-infrared region and lower signal-to-noise ratios reduce their reliability for robust tumor visualization when compared with hemoglobin-based contrast. Importantly, these endogenous contrast mechanisms are highly relevant for gynecological diseases, where vascular remodeling, hypoxia, and inflammation play central roles in conditions such as endometriosis and uterine disorders. The ability of PAI to non-invasively assess these functional parameters provides a strong foundation for disease detection and monitoring.

### 4.2. Exogenous Contrast Agents for Molecular Photoacoustic Imaging

To enhance imaging sensitivity and enable molecular specificity, a wide range of exogenous contrast agents have been developed for photoacoustic imaging. These agents are designed to exhibit strong optical absorption and efficient photothermal conversion, thereby amplifying photoacoustic signals and enabling visualization of weakly absorbing tissues and disease-associated molecular processes. Depending on their composition and functionality, exogenous agents can be broadly classified into nanomaterial-based systems [95,96], small-molecule dyes [26,97], and activatable probes [98,99]. These exogenous contrast agents can be engineered to exhibit red-shifted absorption profiles that minimize interference from intrinsic chromophores, enabling tissue penetration and more selective visualization of cellular and molecular processes beyond conventional anatomical and functional imaging. The development of photoacoustic contrast agents has evolved from clinically approved dyes to engineered nanomaterials and, more recently, to molecularly targeted and activatable probes. To highlight this progression and associated limitations, a comparative summary of representative contrast agents is provided in Table 2.

**Table 2:** Evolution and comparison of exogenous photoacoustic contrast agents.

Category	Representative Agent	Size	Absorption (nm)	Key Advantages	Limitations (Conventional Issues)	Emerging Direction/Improvement	Application	Refs.
First-generation (clinical dyes)	Indocyanine Green (ICG)	~2 nm	~800	FDA-approved, strong NIR absorption	Poor stability, aggregation, rapid clearance, non-specific	Encapsulation, conjugation, activatable probes	In vivo imaging	[100,101]
	Methylene Blue	~2 nm	650–700	Clinically used, easy availability	Low photostability, limited penetration	Modified derivatives, nanoparticle loading	Lymph node imaging	[102,103]
	Evans Blue	<2 nm	~610	Protein binding, vascular imaging	Non-specific distribution, toxicity concerns	Targeted conjugates	Brain imaging	[104,105]
Second-generation (nanomaterials)	Gold nanorods (AuNRs)	20–50 nm	700–900	Tunable absorption, strong PA signal	Potential toxicity, poor biodegradability	Surface modification, biodegradable coatings	Tumor imaging, PTT	[106,107]
	Gold nanorods (AuNRs)	41–45	760	Tunable absorption, strong PA/Raman signal		High sensitivity 17 fM for PAI	Ovarian Cancer	[108]
	Gold nanostars	~35 nm	700–900	High photothermal efficiency	Long-term accumulation	Hybrid/biodegradable systems	Tumor imaging	[109]
	Prussian Blue NPs	20–170 nm	~700	Biocompatible, strong absorption	Limited targeting specificity	Functionalization, exosome coating	Brain tumor imaging	[110,111]
	Polydopamine NPs	~200 nm	Broad absorption from UV to NIR	Biocompatible, strong absorption	Signals are not strong as metallic nanoparticles	Biodegradable with wide absorption	Endometriosis treatment	[112]
Hybrid systems	Exosome–Prussian Blue	~70 nm	700–750	Improved targeting, biocompatibility	Complex synthesis	Biomimetic delivery systems	Tumor imaging	[113]
	BSA-Cerium oxide-ICG	~28 nm	~790	Enhanced stability of ICG	Still partially non-specific	Targeted protein-based systems	Endometriosis	[114]
	Silica shell coated AuNRs	52.75 m	780	Enhanced stability and photothermal conversion efficiency	Still partially non-specific	Targeted Nanoformulation via EPR	Endometriosis PTT	[16]
	Iron oxide- NIR-830 dye	22 nm	820	Targeted imaging probe	Time consuming	Dual imaging modality with high- resolution and specificity	Ovarian cancer	[115]
	Copper sulfide nanodisk/nanoprism	6 and 26	1145 and 1098 nm	Separation between tumor and healthy tissues		Shape-dependent PA signal intensity	Ovarian cancer	[116]
Emerging small-molecule & activatable probes	BODIPY derivatives	<2 nm	700–1000	Tunable structure, high specificity	Limited clinical validation	Enzyme-responsive, ratiometric probes	Molecular imaging/PTT/PDT	[117,118,119]
	Aza-Bodipy	<2 nm	750–1050	Tunable structure	Limited clinical validation	Photostability and thermal conversion	Tumor imaging/PDT/PTT	[120]
	NIR dyes (NIRb14, cyanines)	<2 nm	~800	Strong NIR absorption, easy modification	Photobleaching, off-target signal	Activatable probes (Metalloprotease, hypoxia)	Tumor imaging, PTT	[121]
	Perylene diimide (PDI)	~50–70 nm	400–800	High photostability	Limited water solubility	Functionalized derivatives	Brain imaging/tumor imaging/PTT	[122,123]

#### 4.2.1. Nanomaterial-Based Contrast Agents

Nanomaterial-based contrast agents represent one of the most extensively studied classes of exogenous probes for photoacoustic imaging due to their tunable optical properties, high absorption cross-sections, and versatility in surface functionalization. Among these, gold nanoparticles (AuNPs) have attracted significant attention owing to their localized surface plasmon resonance (LSPR), which enables precise tuning of optical absorption across the visible and near-infrared regions through control of particle geometry [124,125]. A myriad of gold nanostructures, including gold nanorods (AuNRs), gold nanocages (AuNCs), gold nanoshells (AuNShs), and gold nanostars (AuNSts) had been synthesized, offering tunable optical windows from 530 to beyond 1000 nm depending on their anisotropic structure for PAI applications [124].

**Figure 5:**
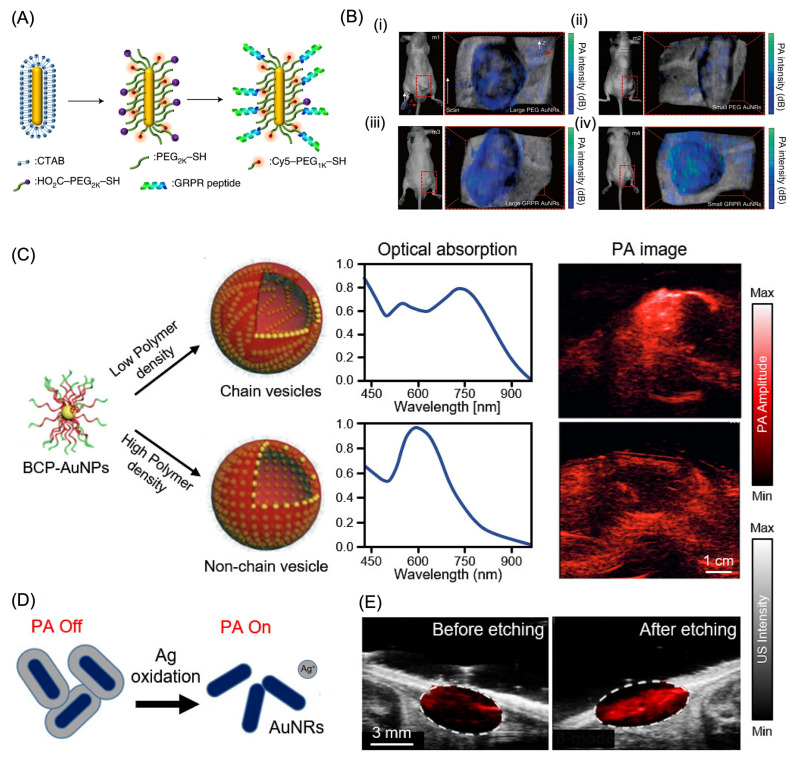
Gold nanoparticle based exogenous contrasting agents for PAI. (**A**) Schematic representation and (**B**) representative photographs of photoacoustic (PA) imaging in tumor-bearing mice (m1–m4) following administration of large and small AuNR formulations. Panels (**i**,**ii**) show PA images of non-targeted large and small AuNRs, respectively, while panels (**iii**,**iv**) display PA images of GRPR-targeted large and small AuNRs. The color-coded PA signal maps are superimposed onto corresponding ultrasound images to provide anatomical context. Reproduced with permission from [127]. Copyright 2019, Nature publishing group. (**C**) Schematic depiction of the preparation of BCP-AuNP vesicles, along with their optical absorption characteristics and contrast-enhanced photoacoustic (PA) images. Yellow dots denote individual nanoparticle building blocks packed within the vesicles, whose spatial organization differs between chain and non-chain structures. Reproduced with permission from [130]. Copyright 2015, Wiley Publishers. (**D**) Illustration of the photoacoustic signal modulation in the presence and absence of a silver (Ag) coating. (**E**) Combined PA and ultrasound (US) images acquired before and after silver etching. Abbreviations: PA, photoacoustic; US, ultrasound; BCP, block copolymer; AuNP, gold nanoparticle; AuNR, gold nanorod; Ag, silver. Reproduced with permission from [131]. Copyright 2018, American Chemical Society.

Among AuNPs, gold nanorods (AuNRs) are among the most widely investigated exogenous photoacoustic contrast agents due to their straightforward synthesis and the ability to precisely tune their optical absorption into the near-infrared region by controlling aspect ratio [126,127]. Chen and co-workers reported the development of ultra-small AuNRs [(8 ± 2) nm × (49 ± 8) nm] with dimensions significantly reduced relative to conventional AuNRs, yet maintaining comparable aspect ratios (Figure 5A) [126]. These miniaturized structures exhibited absorption extending into the NIR-II window (1000–1700 nm) and produced markedly stronger photoacoustic signals than their larger counterparts. Modeling studies attributed this enhancement to a size-dependent increase in surface-to-volume ratio, revealing a quadratic dependence of photoacoustic amplitude on this parameter when optical absorption is normalized across nanorods of different sizes. In vivo experiments demonstrated a 4.5-fold increase in PA signal within tumor tissues following AuNR administration, underscoring the influence of structural properties of nanorods on their optical absorption behavior. Comparative analysis of small and large AuNRs in tumor-bearing mice revealed that both nanoparticles achieved tumor targeting when conjugated with GRPR-binding peptides and Cy5 dyes. After 24 h, non-targeted large AuNRs produced stronger PA signals than smaller counterparts, likely due to tumor heterogeneity (Figure 5B(i,ii)). In contrast, targeted small AuNRs generated higher PA signal intensity compared to larger targeted nanorods, emphasizing the critical role of nanoparticle size in optimizing targeting efficiency and PA imaging performance (Figure 5B(iii,iv)). Despite improved tumor retention, comparable accumulation in clearance organs such as the liver, kidney, and spleen was observed, reflecting limitations associated with reticuloendothelial system uptake.

Beyond tumor imaging, AuNRs have been applied in several vascular and inflammatory imaging contexts [106,128]. Their use as tracers for sentinel lymph node mapping has demonstrated improved imaging depth and contrast relative to spherical gold nanoparticles. Targeted AuNRs and gold nanoshells (AuNShs) functionalized against endothelial inflammatory markers, such as ICAM-1, have enabled intravascular PAI of atherosclerotic lesions and vascular inflammation in preclinical models. In addition, polyethylene glycol-modified hollow AuNShs and nanocages (AuNCs) have been employed for cerebral vasculature imaging following systemic administration, yielding significantly enhanced vessel visibility compared to endogenous contrast alone [128].

Beyond conventional designs, advanced strategies have been developed to further improve functionality. These include gas-generating AuNPs that amplify acoustic signals, hybrid structures that integrate imaging and therapeutic capabilities, and surface-engineered systems that enable targeted or activatable imaging. These AuNPs have been engineered to amplify both ultrasound and photoacoustic signals through laser-triggered nitrogen release, achieved by conjugating azide-containing compounds onto polymer-coated gold surfaces [129]. Upon near-infrared excitation, these nanoparticles act as photocatalysts, generating nitrogen gas that enhances acoustic contrast. Despite these advantages, challenges related to long-term biocompatibility, biodistribution, and clearance remain important considerations for clinical translation.

AuNRs with a longitudinal absorption peak near 760 nm enabled contrast-enhanced PAI of ovarian cancer, allowing clear visualization of MDA-435S xenograft tumors in vivo [108]. To further amplify photoacoustic output, gold nanoparticles were assembled into chain-like vesicular structures using block copolymer templates. These chain vesicles exhibited enhanced optical absorption, generating strong photoacoustic signals at 780 nm and producing an approximately eightfold signal increase compared with non-assembled gold nanoparticles following subcutaneous administration in mice (Figure 5C) [130]. Surface-engineered AuNPs have also enabled activatable photoacoustic contrast mechanisms. Ag-AuNR hybrid nanoparticles were designed with a silver shell that suppressed the photoacoustic signal originating from the AuNR core (Figure 5D) [131]. Upon exposure to ferricyanide, the silver layer was selectively removed, releasing silver ions and unmasking the photoacoustic signal from the underlying AuNRs. This activation produced strong photoacoustic contrast at 750 nm while simultaneously delivering potent antibacterial activity exceeding 99.99%. In vivo PAI before and after silver etching confirmed real-time silver ion release through marked changes in photoacoustic signal intensity (Figure 5E).

In addition to gold-based systems, other plasmonic nanomaterials, including silver and alloyed nanoparticles, have been explored to enhance optical absorption and photoacoustic performance. For example, Ag–Au alloy nanostructures combine the strong plasmonic response of silver with the chemical stability of gold, resulting in improved signal generation while maintaining biocompatibility [132]. In whole-body in vivo mouse imaging, these porous alloyed particles generated substantially higher photoacoustic signals than standard AuNPs, with reported enhancements of approximately 2.7-fold. Beyond Ag–Au alloy systems, other noble-metal pairings, including Pt–Ag and Pd–Pt configurations, have also been explored to fabricate plasmonic architectures with tunable optical properties for PAI applications [133].

Beyond metal nanoparticles, inorganic dye-based nanomaterials such as Prussian blue nanoparticles (PBNPs) have emerged as versatile platforms for multimodal photoacoustic contrast and for photothermal applications [110,111,113]. Building on this concept, size-controlled photomagnetic PBNPs have been synthesized by Dumani et al. using superparamagnetic iron oxide nanoparticles as sacrificial templates [110]. These nanocubes exhibit strong magnetic responses along with pronounced optical absorption near 700 nm, enabling their use as stable, dual-function contrast agents for both magnetic resonance imaging and photoacoustic tomography in vivo. Meghan and colleagues recently reported the development of PBNPs coated with exosomes derived from U-87 glioblastoma cells as a targeted diagnostic platform for PAI of glioblastoma [113]. Owing to their high photothermal conversion efficiency, these exosome-coated nanoparticles were also evaluated for photothermal therapy, resulting in approximately 50% tumor growth inhibition in both in vitro and in vivo models. PAI studies further demonstrated the superior tumor-targeting capability of the exosome-coated PBNPs compared with PEGylated and RGD-functionalized counterparts.

Collectively, nanomaterial-based probes provide a versatile platform for enhancing photoacoustic imaging through tunable optical properties and multifunctional design, although further optimization is required to address translational challenges.

#### 4.2.2. Carbon Nanotube-Based Contrast Agents

Carbon nanotubes (CNTs) have emerged as promising exogenous contrast agents for PAI owing to their strong and broadband optical absorption in the visible and near-infrared regions, which enables high-contrast imaging with improved penetration depth and spatial resolution [134,135,136]. Single-walled carbon nanotubes (SWNTs) generate robust photoacoustic signals even at low laser fluence due to efficient NIR light absorption and thermal confinement [135,137]. Their photoacoustic performance can be dramatically enhanced through molecular functionalization; for example, conjugation with indocyanine green (ICG) increases optical absorbance by up to 20-fold at ~780 nm and yields a ~300-fold improvement in detection sensitivity compared with unmodified SWNTs in vivo imaging study [135]. At this wavelength, background tissue absorption is minimal, further improving signal-to-noise ratios. Notably, ICG exhibits optical absorption efficiencies approximately 7-fold higher than SWNTs and ~8500-fold higher than commercial gold nanorods on a per-weight basis, underscoring the strong photoacoustic sensitization achieved through SWNT–ICG hybrid systems.

Beyond signal enhancement, CNTs offer substantial versatility for targeted, multimodal, and theranostic photoacoustic applications [134]. Gold-plated CNTs, or golden carbon nanotubes (GNTs), integrate the plasmonic properties of gold with the lightweight, hollow cylindrical structure of CNTs, resulting in exceptionally high NIR contrast (up to ~102-fold enhancement), efficient photothermal conversion, and low material dose requirements [138]. These properties have enabled combined photoacoustic and photothermal tumor imaging and ablation with minimal observed toxicity in preliminary studies. CNTs can also be functionalized for molecular targeting, as demonstrated by RGD-conjugated SWNTs that selectively bind αvβ3 integrins and increase tumor photoacoustic signal by 8-fold in glioma xenograft models [135]. Antibody [139], hypoxia [140], and inflammation-targeted CNTs have further enabled sensitive imaging of early tumors [141], atherosclerotic plaques [142], and disease-associated immune cell infiltration [143] using PAI. In addition to oncology, CNT-based agents have been applied to vascular and lymphatic mapping, including sentinel lymph node imaging. They can act as nanoscale photoacoustic emitters capable of generating high-frequency, broadband ultrasound for imaging, drug delivery, and gene transfection [138,144]. Collectively, these studies highlight CNTs as multifunctional PAI platforms that combine high sensitivity, molecular specificity, and therapeutic potential. However, like other nanomaterials, concerns regarding long-term biocompatibility, persistence, and clearance remain key challenges for clinical application.

In addition to CNTs, other carbon-based nanomaterials such as graphene and graphene oxide (GO), carbon dots, and fullerene derivatives have also been explored for photoacoustic and theranostic applications [145]. Graphene-based materials exhibit broad NIR absorption and large surface area for drug loading, while carbon dots offer tunable optical properties and fluorescence–photoacoustic dual imaging capabilities [146,147]. Fullerene derivatives further contribute to photodynamic and theranostic applications due to their unique electronic structures [148]. Incorporating these materials highlights the broader potential of carbon-based platforms in photoacoustic imaging.

While nanomaterial-based and carbon-based contrast agents offer significant advantages in terms of signal amplification and functional versatility, their ultimate impact lies in enabling molecularly specific and disease-relevant imaging. By integrating targeting ligands, responsive elements, or therapeutic components, these systems extend PAI beyond structural imaging toward functional and molecular diagnostics. Such capabilities are particularly relevant in complex diseases characterized by vascular remodeling, inflammation, and microenvironmental changes. In gynecological disorders, including endometriosis and ovarian pathologies, these processes play a central role in disease progression. Therefore, the development of targeted and activatable nanomaterial-based probes provides a promising strategy for improving diagnostic sensitivity and enabling image-guided therapy.

### 4.3. Organic Dyes as Exogenous Contrast Agents

Organic dyes, which can absorb light in NIR-I and NIR-II, are widely used as exogenous contrast agents for PAI and for various biomedical applications [149,150,151]. Clinically approved dyes such as methylene blue (MB), Evans blue (EB), and indocyanine green (ICG) exhibit strong absorption in the visible and near-infrared (NIR-I) region and have been widely employed for contrast-enhanced PAI in applications including lymphatic imaging and vascular mapping. Their relatively low fluorescence quantum yield enables efficient photothermal conversion, thereby enhancing photoacoustic signal generation. While clinically approved dyes such as MB, EB, and ICG provide strong optical absorption and established safety profiles, their performance is often limited by rapid systemic clearance, photobleaching, and non-specific distribution, which can reduce imaging sensitivity and temporal stability in vivo.

#### 4.3.1. Organic Dye-Based Contrast Agents in the NIR-I Window (650–800 nm)

Small-molecule optical dyes have been among the earliest and most widely used contrast agents for lymphatic PAI due to their strong absorption in the visible and near-infrared regions of the spectrum. In rat models, local forepaw injection of MB enabled clear visualization of sentinel lymph nodes (SLNs), yielding approximately a twofold increase in photoacoustic signal intensity relative to control images when excited at 635 nm [152]. Comparable signal enhancement (~2×) was also observed in SLNs following administration of EB, indicating that blue dyes with high visible absorption can effectively accumulate in lymphatic structures and generate measurable photoacoustic contrast [153].

Beyond free dyes, stimulus-responsive formulations have been developed to enable dynamic control of photoacoustic signal generation. For example, dye-loaded microbubbles incorporating MB have been used for combined photoacoustic and ultrasound imaging, with signal intensity in both modalities scaling with microbubble concentration [154]. Notably, exposure to high-intensity ultrasound pulses induces microbubble rupture, resulting in a pronounced (~2.5-fold) increase in photoacoustic signal. This acoustically triggered enhancement highlights the potential for externally controlled activation of photoacoustic contrast. Although stimulus-responsive systems such as microbubble-based platforms enable externally controlled signal amplification, their stability and reproducibility under physiological conditions remain challenges for consistent in vivo applications.

In addition to visible-absorbing dyes, near-infrared chromophores such as indocyanine green (ICG) have become central to contrast-enhanced PAI owing to their strong absorption in the NIR-I window and improved tissue penetration [155,156]. ICG enables clear delineation of lymphatic vessels from surrounding blood vasculature, with reported photoacoustic signal enhancements of approximately 4.3-fold in vivo [157]. Furthermore, its higher fluorescence quantum yield compared to MB and EB allows simultaneous acquisition of fluorescence signals, enabling dual-modal photoacoustic and fluorescence imaging in applications such as lymphatic mapping and organ-specific imaging.

**Figure 6:**
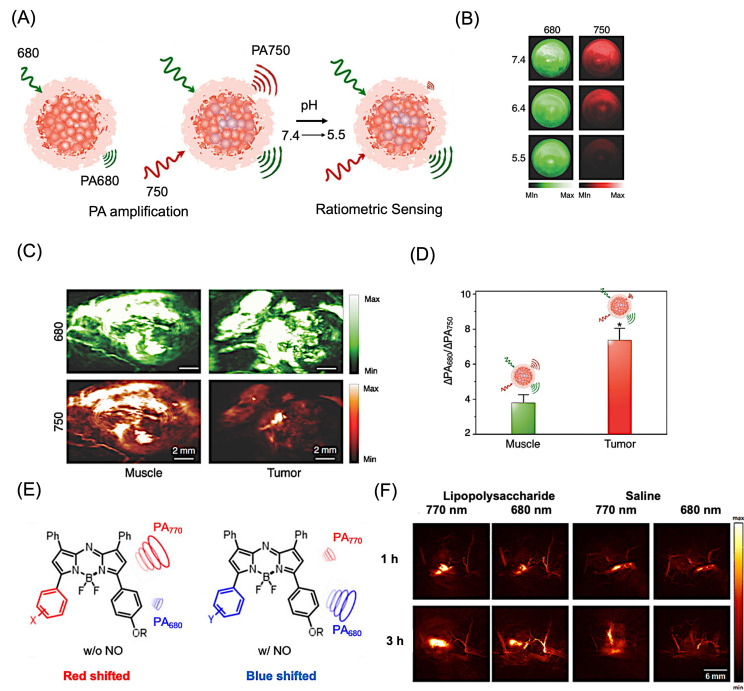
Ratiometric PAI using organic dyes. (**A**) Schematic illustration of the doping-induced photoacoustic (PA) signal amplification and the underlying pH-sensing mechanism. (**B**) PA images of the SON50 solution at different pH conditions (7.4, 6.4, and 5.5). Ratiometric imaging was performed using a pulsed laser tuned to 680 nm and 750 nm. (**C**) PA images and (**D**) corresponding ratiometric signals (ΔPA_680_/ΔPA_750_) obtained from muscle and tumor tissues following local administration of SON50 (10 μL, 10 μg mL^−1^). Representative PA maximum intensity projection (MIP) images in the coronal view are shown. Reproduced with permission from [158]. Copyright 2016, Wiley Publishers. (**E**) Schematic for the signal switching for an NO-activated PA Probe. (**F**) Representative photoacoustic (PA) images demonstrating the response of APNO-5 to endogenous nitric oxide (NO) in a murine LPS-induced inflammation model. PA images were obtained from the mouse flank after subcutaneous administration of LPS or saline, followed by injection of APNO-5 (68 μg kg^−1^). APNO-5 and its transformed product (tAPNO-5) were selectively imaged at 770 nm and 680 nm, respectively. Reproduced with permission from [159]. Copyright 2018, American Chemical Society.

Beyond static contrast enhancement, organic nanostructures have enabled PAI of dynamic biological responses through activatable and environment-sensitive designs. For instance, a pH-responsive nanoprobe based on a semiconducting oligomer–BODIPY system exhibited ratiometric photoacoustic responses under acidic tumor microenvironments, generating distinct signals at 680 and 750 nm in HeLa tumor models (Figure 6A,B) [158]. When administered to HeLa xenograft tumors in mice, the nanoprobes generated tumor-specific PA contrast due to the acidic tumor microenvironment, as revealed by differential signals at excitation wavelengths of 680 and 750 nm in a ratiometric way (Figure 6C,D). Similarly, nitric oxide (NO)-responsive nanoprobes demonstrated a characteristic absorption shift from 770 nm to 680 nm upon interaction with NO, enabling multispectral photoacoustic imaging of inflammation in vivo (Figure 6E) [159]. Multispectral PAI before and after nanoprobe administration in a lipopolysaccharide (LPS)-induced inflammation mouse model confirmed probe activation, producing a significant PA signal enhancement relative to controls (Figure 6F). These activatable systems enable spatial mapping of biochemical processes, providing insight into tumor microenvironment and inflammatory signaling. Despite their high specificity, activatable probes often require complex molecular design and careful calibration of signal response, which may limit their scalability and broader translational applicability.

#### 4.3.2. Organic Dye-Based Contrast Agents with Red-Shifted NIR Absorption (800–1000 nm)

Naphthalocyanine-based agents are near-infrared chromophores with tunable absorption in the 800–1000 nm range, enabling deeper tissue penetration and stronger photoacoustic signals than conventional NIR-I dyes [160]. Their feasibility as in vivo contrast agents has been demonstrated, particularly for gastrointestinal imaging in mice [161]. However, their large, hydrophobic structures can lead to aggregation and limit stability and biocompatibility. To overcome these limitations, derivatives with tunable absorption have been developed for multicolor imaging. For instance, dyes with absorption maxima at 707 nm and 860 nm enabled dual-color PAI and clear lymphatic mapping in vivo [153], although spectral overlap and signal unmixing remain challenges [53]. Nanostructured formulations have further extended their use to tumor imaging and photothermal therapy. In 4T1 models, these systems provided strong photoacoustic contrast and effective photothermal [162]. Naphthalocyanine-loaded nanodroplets have also been used for HIFU-mediated tumor ablation, enabling combined photoacoustic and ultrasound-guided therapy [163]. Encapsulation in perfluorohexane supports strong absorption near 850 nm and effective in vivo imaging, with studies showing enhanced cavitation, increased tumor cell death, and suppressed tumor growth. Despite these advances, system complexity and reliance on external stimuli may limit clinical translation.

#### 4.3.3. Organic Dyes as Contrast Agents with High NIR Absorption (>1000 nm)

**Figure 7:**
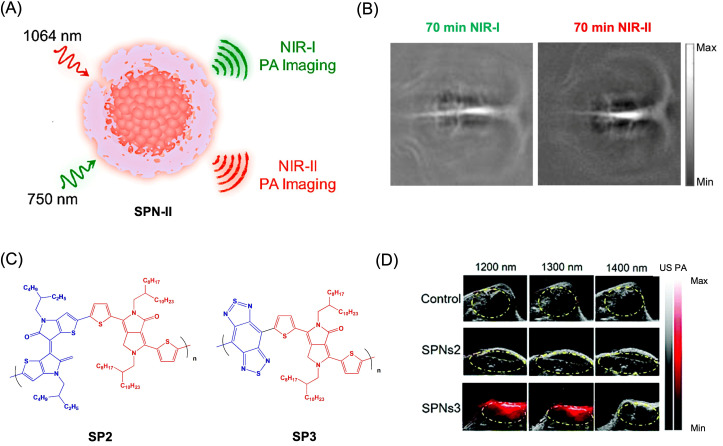
NIR-II organic probes for photoacoustic imaging. (**A**) Schematic illustration of SPN-II nanoparticles for NIR-II PAI applications. (**B**) Representative PA images of the rat cortex acquired 70 min after intravenous administration of SPN-II, recorded at 750 and 1064 nm. SPN-II was injected via the tail vein at a dose of 1.8 mg per rat (n = 3). Reproduced with permission from [164]. Copyright 2017, American Chemical Society. (**C**) Chemical structure of the semiconducting polymer materials used for NIR-II imaging. (**D**) PA/ultrasound co-registered images of tumors without SPNs2–3 injection (control) and after intratumoral injection of SPNs2–3 (50 μL, 500 μg mL^−1^). Dashed circles indicate the tumor region. Reproduced with permission from [166]. Copyright information 2019, RSC publishing group.

Semiconducting polymer nanoparticles (SPNs) are promising platforms for NIR-II photoacoustic imaging due to their donor–acceptor architectures, which enable tunable, red-shifted absorption and improved imaging depth. However, their biodegradability and in vivo clearance remain important considerations for clinical translation. Jiang et al. developed SPNs with one donor and two acceptor units, exhibiting dual absorption at ~750 nm and ~1100 nm (Figure 7A) [164]. In rat brain imaging, NIR-II excitation (1064 nm) produced ~1.5-fold higher signal-to-noise ratio than NIR-I (Figure 7B), highlighting the advantage of longer wavelengths, although increased system complexity may limit broader application. To address clearance, metabolizable SPNs have been designed to degrade into ultra-small (~1 nm) fragments, enabling efficient removal via phagocytic pathways while maintaining strong absorption near 1079 nm for deep imaging [165]. Building on this, SPNs with broad NIR-II absorption (1079–1300 nm) achieved ~2-fold signal enhancement in 4T1 tumor models and enabled high-resolution imaging of tumor and vasculature (Figure 7C,D) [166]. Similarly, optimized two-acceptor SPNs provided signal-to-noise ratios exceeding 20 for cortical imaging at depths approaching 1 mm through the intact skull [167].

Overall, SPNs offer tunable optical properties, improved imaging depth, and high signal fidelity, but challenges in material design, safety, and regulatory approval must be addressed for clinical translation.

### 4.4. Comparative Summary of Preclinical and Clinical Studies Using PAI

While significant progress has been achieved in the development of photoacoustic imaging systems and contrast agents, most reported studies remain at the preclinical stage, primarily involving small animal models and phantom systems. In contrast, clinical investigations are still relatively limited but demonstrate promising potential for translation, particularly in vascular and breast imaging applications. To provide a clearer overview of the translational status and methodological approaches, representative studies discussed in this review are summarized in Table 3, highlighting their classification as preclinical or clinical investigations.

## 5. Quantitative and Dynamic Photoacoustic Imaging Techniques

Dynamic and functional photoacoustic imaging techniques extend the capabilities of conventional PAI beyond structural visualization, enabling quantitative assessment of tissue perfusion, vascular dynamics, and microenvironmental changes. Among these, dynamic contrast-enhanced photoacoustic imaging (DCE-PAI) tracks the uptake and clearance of exogenous contrast agents, providing insight into tissue perfusion and vascular permeability. However, quantitative interpretation of DCE-PAI data can be influenced by factors such as tissue heterogeneity and contrast agent kinetics, which may complicate standardization across studies.

For example, DCE-PAI using indocyanine green (ICG) has been applied to monitor vascular remodeling during wound healing [168]. Time-resolved imaging revealed slower contrast clearance immediately after injury, followed by progressively faster wash-out at later stages, reflecting improved vascular function and tissue recovery. Quantitative analysis of contrast kinetics demonstrated significant differences between early and late healing phases, highlighting the ability of DCE-PAI to capture dynamic physiological changes. Notably, imaging at a single wavelength (800 nm) provided sufficient signal-to-noise for perfusion assessment, simplifying data acquisition without the need for spectral unmixing.

In addition to perfusion imaging, functional PAI approaches have been combined with stimuli-responsive nanomaterials to enable dynamic signal modulation. For instance, thermoresponsive polymer systems incorporating plasmonic or semiconductor nanostructures exhibit temperature-dependent changes in photoacoustic signal intensity [169]. These systems undergo structural transitions near their lower critical solution temperature (LCST), leading to enhanced nanoparticle clustering and increased optical absorption. Such behavior enables controlled amplification of photoacoustic signals and has been explored for imaging-guided photothermal therapy and tumor ablation. While stimuli-responsive systems enable dynamic signal modulation, their dependence on precise environmental conditions may limit reproducibility in complex biological settings.

Collectively, these approaches demonstrate the potential of dynamic and functional PAI techniques to provide quantitative insight into tissue physiology and treatment response. Integration of contrast-enhanced and stimuli-responsive strategies may further enable real-time monitoring of disease progression and therapeutic efficacy in complex biological systems.

## 6. Advantages of PAI Compared with Other Imaging Modalities

PAI is a hybrid modality that uniquely combines optical excitation with ultrasonic detection, thereby overcoming key limitations of conventional optical and clinical imaging techniques. Unlike traditional optical methods that rely on weakly penetrating ballistic photons, PAI detects ultrasound waves generated by optical absorption, allowing high-resolution imaging at depths far beyond the optical diffusion limit. Because acoustic waves scatter minimally in soft tissue, PAI preserves spatial resolution even at depths of several millimeters to centimeters beneath the surface. In the context of women’s health, these advantages are particularly relevant for imaging gynecological conditions such as endometriosis, uterine disorders, cervical cancer, ovarian lesions, and pregnancy-related complications.

PAI is inherently sensitive to optical absorption, making it exceptionally powerful for molecular and functional imaging [170]. By selecting excitation wavelengths matched to specific absorption spectra, PAI can differentiate a wide range of endogenous chromophores, including hemoglobin, melanin, lipids, and water, thereby enabling anatomical, functional, metabolic, and histological imaging without ionizing radiation. The use of exogenous absorbers, including organic dyes, nanoparticles, and genetically encoded probes, further enhances sensitivity and enables deep-tissue molecular imaging under clinically safe laser exposure limits. These capabilities are especially valuable in gynecological diseases where vascular remodeling, hypoxia, and inflammation play central roles, such as in endometriosis, tumor angiogenesis, and placental dysfunction.

Compared with established clinical modalities, PAI offers a unique balance of penetration depth, spatial resolution, and contrast [35,36]. MRI provides excellent soft tissue contrast but suffers from long acquisition times and limited temporal resolution. PET and SPECT enable high sensitivity but are constrained by poor spatial resolution and reliance on ionizing radiation, limiting longitudinal studies. X-ray CT similarly employs ionizing radiation and lacks molecular specificity, while conventional ultrasound imaging provides limited contrast beyond vascular structures [171,172]. PAI bridges these gaps by delivering high-resolution, non-invasive imaging with rich optical contrast at clinically relevant depths.

The advantages of PAI are increasingly supported by translational and early clinical investigations, particularly in oncology [35]. Studies using prototype PAI systems for breast imaging have demonstrated the ability to visualize tumor-associated vascular patterns and reduced hemoglobin oxygenation relative to surrounding healthy tissue, enabling differentiation between benign and malignant lesions and improved lesion delineation [173]. Multispectral imaging implementations have further enabled discrimination of sentinel lymph node metastases in melanoma by separating melanin-derived signals from hemoglobin absorption, addressing a major challenge in conventional nodal assessment. Beyond breast and melanoma applications, PAI-based analyses of hemoglobin oxygenation and lipid content have shown promise in distinguishing malignant from benign tissue in prostate, thyroid, and ovarian specimens [170,174]. Together, these findings underscore the growing potential of PAI as a complementary clinical imaging modality capable of non-invasive functional and molecular characterization of disease. Importantly, unlike many conventional modalities, PAI can be adapted for transvaginal, endocavity, and intraoperative imaging, making it particularly suitable for localized evaluation of pelvic organs and real-time surgical guidance.

An additional advantage of PAI is its scalable resolution, which can be tuned by adjusting the detected acoustic frequency, enabling multiscale imaging from cellular and tissue levels to whole-organ and small-animal imaging using the same optical contrast mechanism [175]. This versatility has driven rapid advances in preclinical research and growing clinical translation, including functional brain imaging [113,176], whole-body small-animal imaging [177,178], human organ visualization [17,179], and emerging machine learning-assisted reconstruction techniques [180,181]. Overall, PAI represents a powerful complementary modality that uniquely integrates depth, resolution, and molecular specificity, positioning it as a transformative tool for biomedical research and future clinical applications in women’s health. Table 4 compares PAI with other imaging modalities in terms of resolution, sensitivity, and application for gynecological conditions, including uterine, ovarian, cervical, and pregnancy-related imaging.

## 7. Application of PAI for Gynecological Disorders

Gynecological diseases represent a major global health concern, with some conditions leading to severe complications such as infertility [2]. Disorders such as vaginitis affect nearly 75% of women worldwide at least once in their lifetime, while gynecological cancers—including ovarian and cervical cancers—continue to pose significant clinical challenges. For example, ovarian cancer is diagnosed in approximately 314,000 women globally, and despite advances in treatment, the five-year survival rate remains below 50% [182]. Similarly, cervical cancer accounted for around 604,000 new cases and 342,000 deaths worldwide in 2020, primarily caused by oncogenic strains of human papillomavirus (HPV), particularly types 16 and 18 [183]. These statistics highlight the urgent need for improved diagnostic and therapeutic strategies.

Gynecological diseases are broadly categorized into inflammatory conditions and tumors. Inflammatory disorders such as vaginitis, cervicitis, and endometritis are often caused by infections or hormonal imbalances, whereas tumors include cancers of the cervix, ovary, vulva, and endometrium. Current treatments mainly involve antibiotics, antifungal drugs, surgery, chemotherapy, radiotherapy, targeted therapy, and immunotherapy [184]. However, these conventional treatments face several limitations, including drug resistance, poor targeting ability, high recurrence rates, and systemic toxicity [185,186,187]. In addition, many gynecological tumors remain asymptomatic during early stages, and inadequate screening methods often result in diagnosis at advanced stages [188]. The non-specific distribution of small-molecule chemotherapeutic agents following intravenous administration further reduces drug accumulation at tumor sites while increasing systemic side effects [189]. These challenges highlight the urgent need for minimally invasive diagnostic tools, targeted therapies, and real-time monitoring strategies to improve treatment outcomes while preserving fertility and minimizing off-target side effects.

In this context, advanced imaging technologies play a crucial role in improving early detection and treatment monitoring. Among these, PAI has emerged as a promising non-invasive technique. PAI enables deep-tissue visualization, high spatial resolution, and molecular-level detection of disease biomarkers, making it particularly valuable for early screening and diagnosis, prognostic assessment, and real-time monitoring of therapeutic responses in gynecological disorders. By integrating targeted molecular probes with PAI, it may become possible to detect inflammatory processes and tumors at earlier stages, ultimately improving clinical outcomes for patients with gynecological diseases. While endometriosis has been more extensively studied in the context of photoacoustic imaging, emerging research in cervical, ovarian, and uterine disorders highlights the broader applicability of these approaches across gynecological diseases.

### 7.1. PAI for Uterine and Endometrial Microvessel Disorders

**Figure 8:**
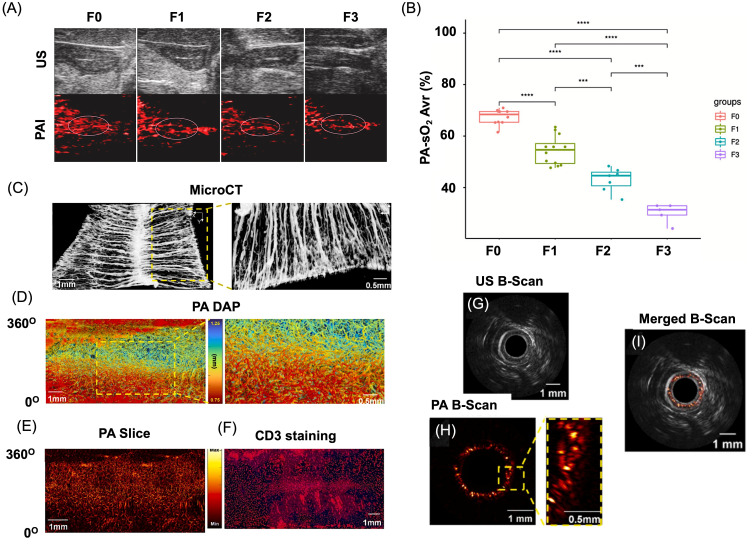
Application of PAI for uterine diseases. (**A**) Representative images from ultrasound and PAI in control rats (F0) and rats with mild (F1), moderate (F2), and severe endometrial fibrosis (F3). The dotted circle shows the area where there is fibrosis. (**B**) Represents the box plot indicating the PA oxygen saturation in each case. *** *p* < 0.001, **** *p* < 0.0001. Reproduced with permission from [190]. Copyright 2024, Elsevier. (**C**–**I**) Microvessels in normal rat endometrium visualized using multiple imaging modalities. (**C**) MicroCT image with corresponding magnified region. (**D**) Photoacoustic DAP image with magnified view. (**E**) Photoacoustic slice at ~300 µm depth. (**F**) CD31 immunostaining of microvasculature. (**G**–**I**) Ultrasonic (US) B-scan, photoacoustic (PA) B-scan with magnified region, and merged US/PA B-scan image. Yellow boxes indicate representative microvessel regions. US: ultrasound; PA: photoacoustic imaging. Reproduced with permission from [191]. Copyright 2024, Elsevier.

Complementing biological and nanomaterial-focused studies, system-level developments have addressed the practical requirements for non-invasive pelvic imaging. Photoacoustic spectroscopy and hybrid ultrasound–photoacoustic platforms designed for abdominal cavity imaging emphasize the importance of illumination geometry, acoustic detection sensitivity, and real-time imaging capability for clinical deployment [27]. Related work in adjacent uterine pathologies, such as intrauterine adhesions, further supports the value of functional photoacoustic metrics, including oxygenation and perfusion, for grading disease severity, suggesting that similar quantitative biomarkers could be adapted for endometriosis assessment [190]. Intrauterine adhesion (IUA) is the secondary reason for infertility in women. In a recent study, Dong et al. showed that endometrial oxygen saturation can correlate with IUA using PAI, overcoming limitations of ultrasound-only diagnosis (Figure 8A) [190]. In this study, IUA was established in a rat model through mechanically induced uterine injury to mimic fibrosis and adhesion formation. The animals were subsequently examined using both PAI and high-frequency ultrasound imaging to compare diagnostic performance. Ultrasound imaging provided anatomical visualization of the uterine structure, while PAI measured hemoglobin-related optical absorption signals to assess vascularity and tissue oxygenation. The results showed that adhesion regions exhibited reduced vascular signals and altered oxygenation patterns, which PAI detected more clearly than by ultrasound alone. Quantitative analysis demonstrated that PAI enabled more sensitive evaluation of adhesion severity and functional tissue changes, highlighting its advantage for non-invasive assessment and monitoring of IUA (Figure 8B).

Endometrial microvessels provide oxygen and nutrients to the embryo, and they are significant in evaluating endometrial receptivity. Endoscopic PAI is used to study such microvessel systems, which enabled real-time, high-resolution visualization of endometrial microvessels and blood perfusion using endogenous hemoglobin contrast, outperforming conventional ultrasound in functional vascular assessment [191]. Figure 8C–I compares endometrial microvascular imaging in normal rats using multiple imaging modalities. The endometrium exhibited a dense, basket-like vascular organization consistent with previously reported anatomical observations. MicroCT imaging following contrast administration provided a macroscopic overview of uterine vasculature; however, fine microvascular structures were not clearly resolved in either the original or magnified views (Figure 8C). In contrast, PAI enabled detailed visualization of the endometrial vascular network, with three-dimensional reconstructions revealing continuous microvessel architecture (Figure 8D). Compared with the 3D datasets, two-dimensional photoacoustic slices displayed partial vessel discontinuity due to limited sectional information (Figure 8E). Histological validation using CD31 staining confirmed the abundance of superficial endometrial microvessels, although detailed structural morphology could not be fully appreciated from histology alone (Figure 8F). Sequential B-scan imaging using endoscopic ultrasound, PAI, and their merged outputs further demonstrated depth-resolved vascular patterns, clearly revealing microvessel distribution within the tissue (Figure 8G,I). Signal analysis indicated that the strongest photoacoustic response originated from a depth of approximately 1.22 mm, corresponding to an effective imaging penetration depth of at least 470 µm.

These findings highlight that PAI-based vascular and functional imaging strategies developed in uterine disorders provide a foundation for broader gynecological applications, including tumor detection and inflammatory disease monitoring. From a functional imaging perspective, multispectral PAI targeting hemoglobin-derived contrast is particularly suitable for assessing vascular remodeling and tissue oxygenation associated with uterine pathologies.

### 7.2. PAI for Endometriosis

Endometriosis (EM) represents one of the most extensively studied models for PAI due to its vascular and inflammatory characteristics, which make it particularly suitable for both diagnostic and theranostic investigations. Endometriosis (EM) is a prevalent gynecological disorder characterized by ectopic growth of endometrial-like tissue, leading to chronic pelvic pain and infertility. Despite its clinical burden, early diagnosis remains challenging [10,114,192]. Conventional imaging modalities such as ultrasound, MRI, and CT suffer from limited sensitivity and poor lesion contrast, particularly for small or superficial lesions, often necessitating invasive laparoscopic confirmation. These limitations highlight the need for sensitive, non-invasive imaging strategies for early detection.

Recent advances in biomedical imaging have introduced new opportunities to improve EM diagnosis. In this context, PAI enables visualization of lesion-associated vascular remodeling through endogenous absorbers such as hemoglobin. However, reliance on intrinsic contrast limits sensitivity for deeply located or weakly vascularized lesions, necessitating the development of enhanced imaging strategies. To overcome these limitations, exogenous contrast agents, particularly nanoparticle-based systems, have been developed to enhance signal strength, spectral tunability, and target specificity. These platforms enable high signal-to-noise lesion visualization and can be engineered to target disease-relevant biomarkers. In addition, nanomaterials such as gold-based systems provide efficient photothermal conversion, enabling image-guided ablation of lesions [12,16,20,112]. This integration of diagnostic and therapeutic capabilities highlights the potential of nanoparticle-enabled PAI as a versatile platform for non-invasive detection and treatment of endometriosis. From a design perspective, the protease-rich inflammatory microenvironment of endometriotic lesions makes activatable probes targeting MMP-2/9 and cathepsins targeting is particularly suitable, enabling selective signal amplification and improved lesion specificity.

#### 7.2.1. PAI for Non-Invasive Detection of Endometriosis

**Figure 9:**
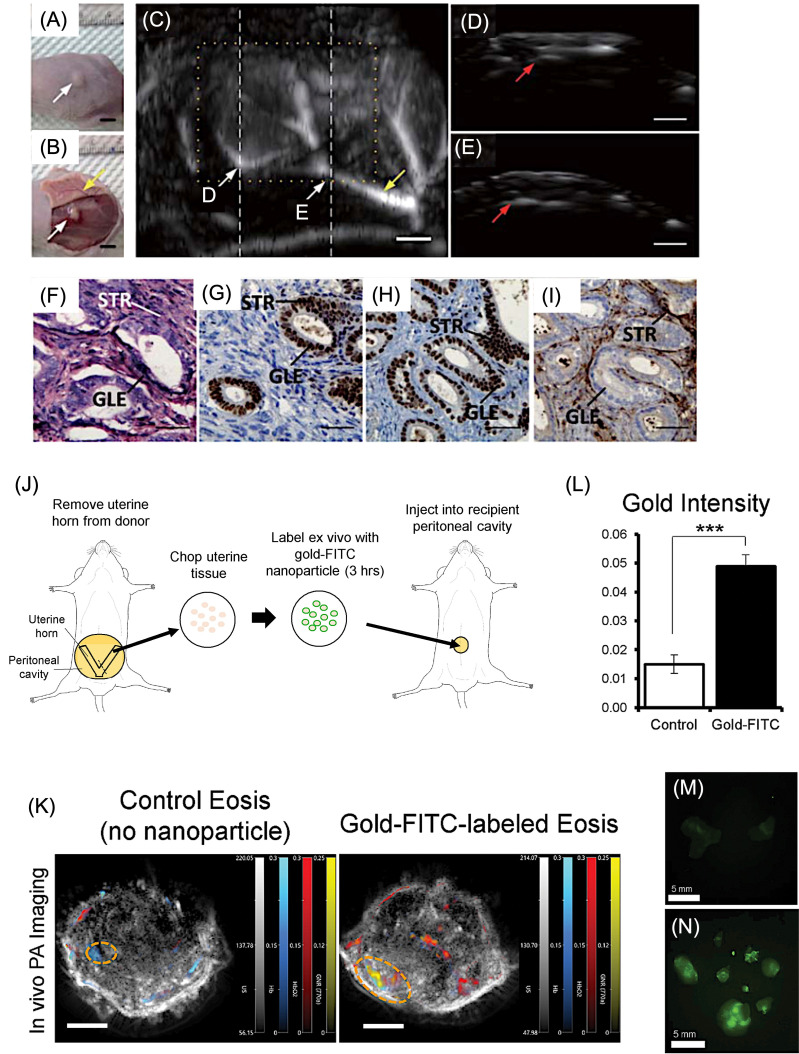
PAI application for endometriosis. (**A**) Positive identification of the endometriosis lesion using PA microscopy. (**A**,**B**) Macroscopic images of the EM lesion (white arrows) before and after anatomical dissection. (**C**) MAP image showing the EM lesion and surrounding blood vessels. Yellow dotted lines indicate the region of interest. White arrows (**B**,**C**) indicate selected regions along the structure used for cross-sectional analysis, while the yellow arrow highlights the region of interest. (**D**,**E**) Corresponding B-scan images from the regions marked in (**B**,**F**) H&E staining. Red arrows in panels (**D**) and (**E**) denote localized signal features in the corresponding enlarged views. (**G**–**I**) Immunohistochemical staining for PR, ER, and CD10. GLE: glandular epithelial cells; STR: stromal cells. Scale bars: (**A**,**B**) 5 mm; (**C**–**E**) 1 mm; (**F**–**I**) 50 µm. Reproduced with permission from [194]. Copyright 2014, Wiley Publishers. (**J**) Schematic of the surgical induction of endometriosis in mice using gold–FITC nanoparticle-labeled uterine tissue. (**K**) Representative in vivo PA images from control mice (no nanoparticles, **left**) and mice with gold–FITC nanoparticle-labeled lesions (**right**), showing PA signals for gold (yellow), Hb (blue), and HbO_2_ (red) four weeks after induction. Orange dashed circles indicate lesions identified by dissection (**left**) or detected by PA imaging and confirmed by dissection (**right**). Scale bar = 5 mm. (**L**) Quantification of gold PA signal intensities from ROIs around endometriosis lesions (mean ± SEM; control n = 4, gold–FITC NP n = 3; *** *p* < 0.001). PA signals were co-registered with B-mode ultrasound images (gray). (**M**,**N**) FITC fluorescence images of control and gold–FITC nanoparticle-labeled lesions after lesion dissection four weeks post-induction. Green fluorescence indicates nanoparticle presence. Scale bar = 5 mm. Reproduced with permission from [195]. Copyright 2022, Springer Publishers.

Recent preclinical studies demonstrate that both endogenous and exogenous photoacoustic contrast mechanisms can be exploited to detect, characterize, and monitor endometriotic lesions [15,193]. Early work using photoacoustic microscopy established the feasibility of imaging endometriosis-associated vascular remodeling in murine models by leveraging endogenous hemoglobin contrast [194]. These studies revealed distinct microvascular and perfusion patterns associated with lesion development, demonstrating the potential of label-free PAI to probe lesion biology. As shown in Figure 9A,B, photoacoustic microscopy (PAM) identified lesion-associated vascular structures, with MAP images highlighting prominent surrounding blood vessels. Corresponding B-scan images (Figure 9D,E), obtained along the lines indicated in Figure 9C, showed strong PA signals originating from the lesion at depths exceeding 1 mm. Histological and immunohistochemical analyses (Figure 9F–I) further confirmed endometriotic glandular and stromal features. However, the limited penetration depth of PAM restricts its applicability to superficial tissues, motivating the development of deeper-tissue PAI approaches.

To overcome sensitivity and depth limitations, contrast-enhanced PAI approaches employing nanoparticles have been introduced [112,195]. Gold nanorod (AuNR)-based labeling approaches have enabled robust visualization of lesions when combined with ultrasound co-registration, facilitating longitudinal imaging and multimodal validation through complementary fluorescence signals. Ryan et al. prepared AuNRs labeled with FITC(fluorescein isothiocyanate) as multimodal imaging agents for endometriosis [195]. A stitch model of endometriosis was established using uterine horn tissue fragments labeled with AuNR–FITC nanoparticles (Figure 9J). Following lesion development, non-invasive whole-body PAI revealed strong gold-associated signals colocalized with oxyhemoglobin and deoxyhemoglobin in nanoparticle-labeled lesions, while no comparable signals were observed in controls (Figure 9K). These findings indicate selective nanoparticle accumulation within highly vascularized endometriosis-like lesions. Quantitative analysis showed a significant increase in gold-derived signals in nanoparticle-labeled tissues compared to controls, while local oxygen saturation remained unchanged (Figure 9L). Post-dissection imaging further confirmed FITC-positive lesions only in nanoparticle-treated mice (Figure 9M,N). Additionally, the combined PA–ultrasound platform enabled early detection and longitudinal monitoring of lesions, highlighting the improved sensitivity of nanoparticle-enhanced PAI over endogenous contrast alone.

Overall, nanoparticle-enhanced PAI significantly improves lesion detectability compared to endogenous contrast alone, particularly for small or deep-seated lesions. These advances highlight the potential of PAI as a non-invasive imaging modality for early detection and characterization of endometriosis.

#### 7.2.2. Endometriosis Treatment Validation Using PAI

**Figure 10:**
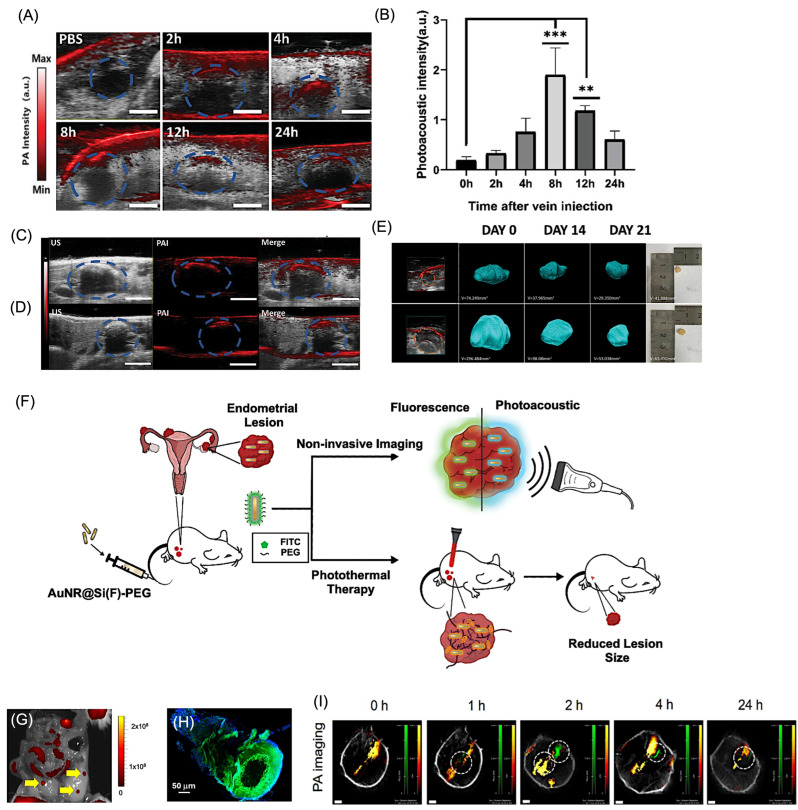
Application of PAI for endometriosis diagnosis and treatment. (**A**) PAI of endometriosis lesions after injection of PBS or PDA@HA at 2, 4, 8, 12, and 24 h. The blue dotted circles indicated the EM lesion and area of interest. (**B**) Quantification of time-dependent PA signal intensity in lesions (*p*-value: 8 h: *** *p* < 0.001, 12 h: ** *p* = 0.006; n = 3 per group). (**C**) Representative US, PAI, and merged images of lesions showing distinct acoustic scattering variation. (**D**) US, PAI, and merged images of lesions without noticeable acoustic scattering variation (scale bar: 4 mm). (**E**) Lesion volumes measured by US–PAI at days 0, 14, and 21 after PBS or treatment, with corresponding photographs of excised lesions (orange dashed circles indicate lesions; n = 4 per group). Reproduced with permission from [112]. Copyright 2024, Wiley Publishers. (**F**) Schematic for the working principle for AuNR@Si(F)-PEG NPs for endometriosis detection and treatment. (**G**) IVIS Spectrum in vivo imaging of a systemic mouse model used to validate endometriosis-like lesions following intravenous injection of AuNR@Si(F)-PEG nanoparticles. (**H**) Fluorescence microscopy images confirming endometriosis lesions after excision from mice. Blue = DAPI; green = FITC. (**I**) Representative in vivo PA images from the endometriosis lesions in mice with AuNR@Si(F)-PEG NPs at different time intervals. The white dotted circles indicate the EM Lesion where the PA signal from AuNR overlapped with HbT. AuNR (green), HbT (orange-red). Reproduced with permission from [16]. Copyright 2025, Wiley Publishers.

Beyond detection, PAI has also been explored as a theranostic platform for endometriosis. Polydopamine-based nanoparticles modified with targeting ligands, such as hyaluronic acid (PDA@HA), have been shown to significantly enhance photoacoustic signal intensity within endometriotic lesions in vivo [107]. Following intravenous administration, time-dependent signal enhancement was observed, with peak accumulation at intermediate time points (Figure 10A,B). Concurrent ultrasound imaging provided anatomical reference, and combined US–PAI improved lesion delineation, particularly for lesions with weak ultrasonic contrast (Figure 10C,D). Quantitative analysis confirmed dynamic nanoparticle accumulation and clearance, supporting optimal imaging windows and improved lesion visualization (Figure 10B). These nanoparticles also enabled image-guided therapeutic assessment, where lesion size reduction was monitored using US–PAI systems (Figure 10E). Collectively, these findings demonstrate that PDA@HA-assisted PAI enables both enhanced imaging and treatment monitoring of endometriotic lesions.

Silica-coated gold nanorods (AuNRs) have been explored as both photoacoustic contrast agents and photothermal transducers for targeted ablation of endometriosis lesions. Kumar et al. developed FITC-labeled AuNR@Si(F)-PEG nanoparticles, enabling combined photoacoustic and fluorescence imaging of lesions (Figure 10F) [16]. Following administration, strong fluorescence signals were observed at lesion sites and confirmed by excision and tissue staining (Figure 10G,H). Non-invasive PAI further revealed strong AuNR-associated signals colocalized with total hemoglobin, indicating selective accumulation in highly vascularized lesions (Figure 10I). Quantitative analysis confirmed enhanced signal intensity compared to controls, while biodistribution studies showed predominant accumulation in the liver and lesions. Owing to their efficient photothermal properties, AuNRs enabled image-guided ablation of lesions, resulting in significant reduction in lesion volume. These findings highlight the potential of PAI for real-time visualization and monitoring of therapeutic response. However, challenges such as thermal dose control, off-target heating, and long-term safety remain. In another study, Rahman et al. showed the use of ICG-conjugated nanoceria for theranostics of endometriosis using PAI [114]. Owing to the anti-inflammation property of nanoceria, a reduced number of lesions were observed in the nanoparticle-treated group compared to the control groups. Moreover, there were no adverse effects for the pregnancy in mice using this nanoparticle as compared with tofacitinib, which showed potential infertility for long-term use.

Collectively, existing studies position PAI as a promising modality for endometriosis research and diagnosis. Endogenous PAI provides valuable insight into lesion vascular physiology, while nanoparticle-enhanced and theranostic approaches expand sensitivity and functional scope. Future progress will depend on improving molecular specificity, standardizing quantitative imaging metrics, and integrating PAI with clinically established ultrasound workflows. With continued advances in contrast agent design, biomarker selection, imaging hardware, and validation in clinically relevant models, PAI has strong potential to evolve into a non-invasive tool for detection, therapy guidance, and longitudinal monitoring of endometriosis.

### 7.3. Cervical Tissue Remodeling and Pregnancy-Related Conditions

The human cervix is a dynamic tissue composed of smooth muscle, epithelial cells, fibroblasts, and blood vessels embedded within a collagen-rich extracellular matrix primarily consisting of type I and type III collagen, elastin, and proteoglycans [196]. During pregnancy, progressive cervical remodeling alters tissue stiffness and length through reorganization of the collagen network, increased hydration, extracellular matrix degradation, and enhanced vascularity. These structural and biochemical changes intensify as delivery approaches, and premature cervical shortening is strongly associated with an increased risk of preterm birth [197]. Evidence suggests that early disruption or disorganization of cervical collagen architecture contributes to abnormal remodeling and may serve as an important predictor of preterm delivery [198]. PAI enables functional assessment of cervical remodeling by quantifying changes in vascularity and oxygenation associated with collagen reorganization, providing complementary information beyond conventional ultrasound-based cervical length measurements.

Yan et al. studied the collagen-to-water ratio (CWR) in cervical tissue compositions excised human samples tissues using PAI [196]. The study compared cervical tissues from pregnant (21–36 years, median 30) and non-pregnant hysterectomy patients (29–58 years, median 42) to evaluate structural and compositional differences using spectroscopic photoacoustic (sPA) imaging and histology. Averaged sPA spectra revealed that non-pregnant cervical tissues exhibited strong collagen-associated photoacoustic peaks within the 1150–1250 nm range, whereas pregnant tissues showed higher signal amplitudes in the water-dominant region (1300–1650 nm), indicating increased tissue hydration. Histological analyses (H&E and Sirius Red staining) confirmed these findings, demonstrating dense, well-organized collagen bundles in non-pregnant samples compared to loosen collagen architecture with edema in pregnant tissues. Quantitative wavelength unmixing showed significantly higher collagen-to-water ratio (CWR) in non-pregnant women (55.0% ± 20.3%) than in pregnant women (18.7% ± 7.5%; *p* = 0.00016). Subgroup analysis of patients under 41 years further validated this difference, supporting pregnancy-associated collagen remodeling and increased water content in cervical tissue. The developed imaging platform has the potential to enable accurate and reliable screening and diagnostic assessment with improved sensitivity and specificity, facilitating early detection of cervical insufficiency and offering a promising approach for predicting the risk of preterm birth. Functional PAI approaches enabling real-time assessment of placental oxygenation and perfusion are particularly valuable for monitoring pregnancy-related complications.

A dual-illumination US/PAI system was developed by Basiji et al. to enhance cervical cancer detection. The study demonstrated that cancerous cervical tissues produced higher photoacoustic signal amplitudes compared to normal tissues, due to increased hemoglobin content and tumor-associated vascularization [199]. Dual-sided light delivery improved optical penetration depth and signal uniformity, enabling more reliable visualization of deep cervical regions. The integrated imaging approach allowed simultaneous anatomical localization and functional assessment of tissue composition. These findings highlight the potential of PAI as a non-invasive modality for early diagnosis and characterization of cervical cancer. The same group studied the biochemical and structural changes in the uterine cervix during murine pregnancy by quantifying collagen and water content [200]. Spectroscopic photoacoustic (sPA) measurements revealed a progressive decrease in collagen-associated signals, accompanied by increased water-related signals, reflecting pregnancy-induced cervical remodeling. These findings were validated by histological staining, which showed collagen disorganization and increased tissue hydration during gestation. The study demonstrated that PAI enables non-invasive, label-free monitoring of cervical softening processes. This approach highlights the potential of PAI for assessing cervical maturation and predicting pregnancy-related complications such as preterm birth.

Spectral PAI was utilized to evaluate age-related collagen remodeling in the reproductive tract using a mouse model of pelvic organ prolapse [201]. In a study by Markel et al. a wavelength-dependent PAI analysis revealed a significant reduction in collagen-associated photoacoustic signals in aged mice compared with young controls, indicating loss of collagen density and structural integrity. Their quantitative spectral unmixing demonstrated decreased collagen-to-water ratios (CWRs) and altered tissue composition consistent with age-associated extracellular matrix degeneration. These photoacoustic findings were corroborated by histological analyses showing disrupted collagen organization and reduced fiber alignment in prolapsed tissues. Collectively, the results demonstrate that spectral PAI enables non-invasive, label-free assessment of collagen degradation and provides a promising tool for monitoring tissue remodeling associated with aging and pelvic organ prolapse. These approaches demonstrate the potential of PAI for early detection and molecular characterization of cervical pathologies, particularly when integrated with targeted contrast agents and multimodal imaging strategies. From a technical standpoint, hypoxia-responsive and angiogenesis-targeted probes are well-suited to capture tumor-associated vascular heterogeneity and oxygenation gradients.

### 7.4. Ovarian Pathologies

**Figure 11:**
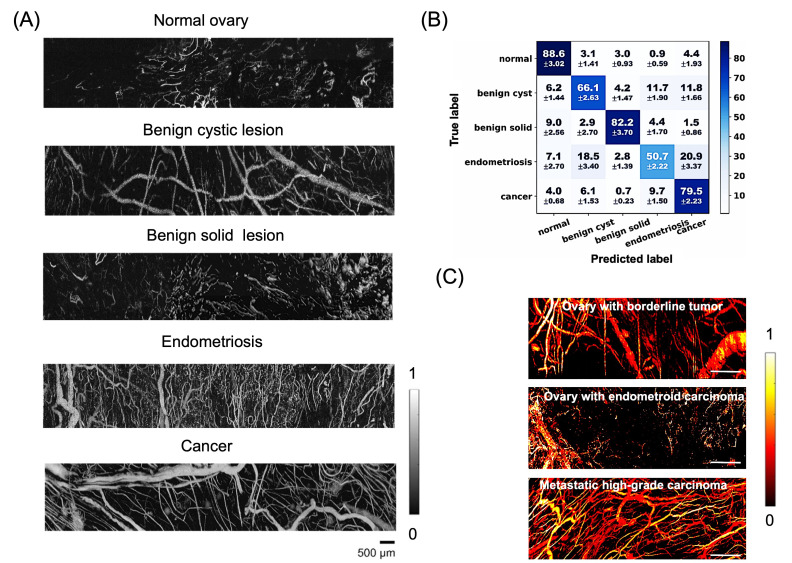
PAI application in ovarian pathologies. (**A**) OR-PAM images of ovarian lesions representing different pathological conditions, displayed with a 40 dB dynamic range. (**B**) Performance of VGN for five-class classification. The confusion matrix represents the average of 100 random train–test splits and is normalized by the number of vascular graphs in each category to report sensitivity (%) for each class. Standard deviations derived from cross-validation are shown below the sensitivities to indicate prediction stability. Reproduced with permission from [203]. Copyright 2026, Elsevier. (**C**) Representative micrographs of ovarian and fallopian tube lesions exhibiting different levels of malignancy. Scale bar: 500 μm. Reproduced with permission from [204]. Copyright 2022, Nature Publishing group.

Diagnosis of ovarian cancer and lesions is challenging due to the heterogeneity in clinical reports [202]. Non-invasive detection techniques, such as US/PAI, can be used for the early detection of such disease conditions. In one of the studies, optical-resolution photoacoustic microscopy (OR-PAM) combined with a vascular graph network was developed for automated classification of ovarian lesions based on microvascular architecture [203]. The study extracted quantitative vascular features, including vessel density, branching patterns, and network topology, from high-resolution photoacoustic images to characterize lesion-associated angiogenesis. As shown in Figure 11A, the vasculature in normal ovaries was very thin and sparsely distributed. But the ovarian lesions showed abnormalities and morphological deformations under different pathological conditions. The graph-based deep learning model achieved accurate differentiation between normal and pathological ovarian tissues by leveraging vascular structural information derived from photoacoustic signals. As shown in Figure 11B, the classification performance of the vascular graph network (VGN) at the individual graph level demonstrates strong diagnostic accuracy across different ovarian tissue types. The model achieved the highest sensitivity and specificity in distinguishing normal ovaries and benign solid lesions. Importantly, the system showed robust capability in differentiating malignant tumors from benign pathologies, which is critical for clinical diagnosis. Analysis of vascular grafts derived from cancerous tissues yielded a sensitivity of 0.795 ± 0.022 and an area under the curve (AUC) of 0.877 (95% CI: 0.859–0.891), indicating reliable cancer classification performance. These results highlight the effectiveness of vascular-feature-based photoacoustic analysis for accurate ovarian lesion discrimination. This work highlights the potential of photoacoustic microscopy integrated with artificial intelligence for non-invasive, label-free ovarian cancer diagnosis and automated lesion classification. In another study, the same research group demonstrated the use of OR-PAM for label-free visualization and measurement of vascular parameters, including vessel density, diameter, branching patterns, and spatial organization [204]. The study demonstrated significant vascular heterogeneity in ovarian lesions compared with normal tissues, reflecting disease-associated angiogenic remodeling (Figure 11C). Quantitative analysis revealed distinct vascular characteristics that allowed differentiation between pathological and healthy reproductive tissues.

A combined ultrasound–photoacoustic (US/PA) imaging protocol was developed for transvaginal imaging of ovarian lesions to enable simultaneous anatomical and functional assessment [205]. The integrated approach combined ultrasound-based structural visualization with photoacoustic detection of hemoglobin-related optical contrast, allowing improved characterization of lesion vascularity. The study demonstrated enhanced visualization of ovarian lesion boundaries and vascular features compared with ultrasound imaging alone. Photoacoustic signals provided additional functional information related to blood content and tissue heterogeneity, facilitating differentiation between lesion types. These results highlight the potential of combined US/PA transvaginal imaging as a non-invasive strategy for improved evaluation and diagnosis of ovarian abnormalities. Like advances demonstrated in endometriosis, nanoparticle-enhanced and multimodal PAI approaches may further improve sensitivity and enable image-guided therapeutic strategies in ovarian pathologies.

Given the deeper anatomical location of ovarian tissues, PACT-based approaches combined with NIR-absorbing contrast agents are more suitable for achieving sufficient imaging depth and sensitivity. Although endometriosis currently represents the most extensively investigated application of PAI in gynecology, emerging studies across uterine, cervical, and ovarian pathologies indicate that these imaging strategies are broadly transferable, and hold promise for diverse clinical applications. A concise summary of representative studies, including model type, comparator modalities, and translational readiness, using PAI for gynecological applications are provided in Table 5.

## 8. Clinical Translation and Integration

PAI is steadily advancing toward clinical implementation, driven by the development of probe-based and intraoperative imaging systems designed for real-time use in surgical and diagnostic procedures [29]. The integration of photoacoustic technology with conventional ultrasound platforms has been particularly impactful, enabling simultaneous acquisition of anatomical, functional, and molecular information, while maintaining the familiarity and efficiency of established clinical workflows. Multimodal combinations with optical, Doppler, and spectroscopic imaging further enhance diagnostic capability by providing complementary tissue contrast and quantitative physiological parameters. Importantly, advances in laser safety standards, portable light-delivery technologies, and regulatory-aligned device engineering have improved the feasibility of clinical adoption by addressing safety, usability, and workflow requirements. Together with the emergence of workflow-compatible handheld and transvaginal probes, these advances position PAI as a promising translational modality for intraoperative guidance, disease diagnosis, and real-time clinical decision-making.

PAI community is currently advancing toward greater standardization, a critical step for ensuring reliable clinical translation and multicenter implementation. The International Photoacoustic Standardization Consortium (IPASC) has established consensus guidelines for technical specifications, data-acquisition parameters, and reporting standards to reduce variability between imaging systems [206]. To address inconsistencies in proprietary data formats, standardized metadata structures and an open-source application programming interface were introduced, enabling conversion into a unified format and facilitating data exchange and cross-site comparison. These initiatives have also accelerated the integration of PAI data into the Digital Imaging and Communications in Medicine (DICOM) framework, thereby improving compatibility with existing clinical imaging infrastructure [180,181,207]. Ongoing efforts include the development of open-source image-reconstruction libraries that enable systematic evaluation of algorithm performance, particularly as artificial intelligence methods become increasingly integrated into PAI data analysis. In parallel, consensus terminology, imaging-biomarker definitions, and image-quality metrics are being established to harmonize communication across research and clinical communities. Furthermore, recognizing the need for reproducible validation, standardized tissue-mimicking phantoms and testing protocols are being developed to provide reliable ground-truth datasets for performance assessment, quality assurance, and system calibration. Although full clinical standardization is still underway, the technical similarity of PAI to ultrasound and its favorable safety profile have already enabled early clinical studies and multicenter investigations. Continued progress will require coordinated regulatory alignment, standardized clinical training programs, and close collaboration among engineers, researchers, and healthcare professionals to ensure safe and consistent clinical adoption.

Several PAI platforms have been translated toward clinical use. Representative systems include multispectral optoacoustic tomography (MSOT) devices such as the Acuity Echo platform, hybrid ultrasound–photoacoustic systems such as the Imagio breast imaging system, and emerging mesoscopic systems including raster-scan optoacoustic mesoscopy (RSOM). In addition, handheld probes and endoscopic photoacoustic devices are under development to enable real-time clinical imaging and image-guided interventions. Table 6 summarizes major clinically investigated PAI systems and their applications.

PAI has shown strong performance in preclinical small-animal studies and is increasingly transitioning toward clinical applications. Several commercial PAI platforms have also emerged to support this transition [208]. Although implementations vary, three primary configurations dominate current clinical investigation according to the anatomical region of interest and diagnostic objective: dual-modal photoacoustic–ultrasound imaging (PAUSI), station-based tomographic PAI, and mesoscopic/microscopic PAI [17,29].

**Figure 12:**
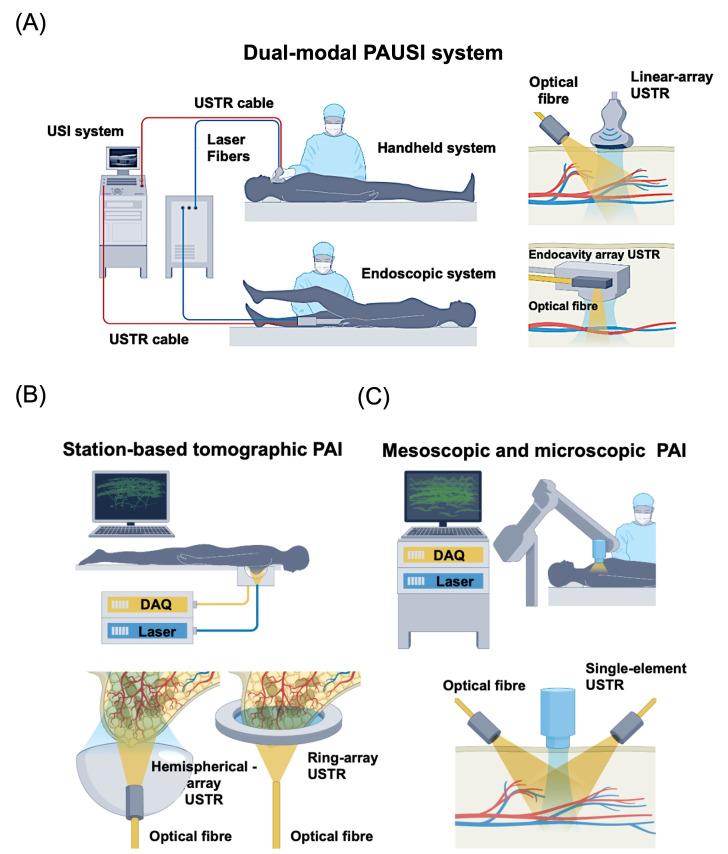
Application of various PAI systems for clinical trials. (**A**) Dual-modal photoacoustic–ultrasound imaging (PAUSI) systems are derived from conventional ultrasound imaging (USI) platforms and are generally implemented in two formats: a handheld configuration employing linear-array ultrasound transducers (USTRs) and an endoscopic configuration utilizing endocavity array transducers. Handheld systems are primarily used for imaging superficial tissues such as the skin, thyroid, breast, and peripheral limbs. In contrast, endoscopic implementations enable imaging of internal regions, including the stomach, vaginal canal, and rectum. (**B**) Station-based tomographic PAI employs hemispherical or ring-shaped ultrasound transducer arrays, which allow efficient detection of photoacoustic signals for tomographic reconstruction. These systems are mainly applied to imaging brain, breast, and limb tissues. (**C**) Mesoscopic and microscopic PAI utilize high-frequency single-element ultrasound transducers, enabling high-resolution imaging. This configuration is particularly suited for examining superficial tissues such as skin. Reproduced with permission from [29]. Copyright 2024, Nature Publishing Group.

Dual-modal PAUSI integrates a conventional ultrasound imaging (USI) system with a portable pulsed laser, allowing simultaneous acquisition of ultrasound and photoacoustic signals (Figure 12A). This approach provides complementary information on tissue structure and blood flow from ultrasound, while adding optical absorption-based contrast from photoacoustics, such as hemoglobin concentration, oxygen saturation, and molecular probe distribution. PAUSI systems can be configured as handheld or endoscopic devices. Handheld systems typically employ linear, phased, or curvilinear ultrasound arrays coupled with optical fiber bundles and are commonly used for imaging superficial tissues, including the skin, thyroid, breast, and limbs. Endoscopic PAUSI systems, incorporating transrectal or transvaginal probes with miniaturized optical fibers, enable imaging of internal organs and facilitate the diagnosis of rectal and gynecological diseases.

Station-based tomographic PAI employs large detection apertures, such as hemispherical or ring-shaped ultrasound transducer arrays, to efficiently capture omnidirectional photoacoustic waves and improve image reconstruction quality (Figure 12B). In typical clinical configurations, the detector array is placed beneath the imaging platform and integrated with a pulsed-laser and synchronized ultrasound-acquisition electronics. A motorized stage allows scanning over larger ROIs, expanding clinical applicability. These systems provide relatively deep tissue imaging in humans (up to ~5 cm) while maintaining high spatial resolution, and have been applied to organs such as the brain, breast, and peripheral limbs.

Mesoscopic and microscopic PAI utilize high-frequency single-element ultrasound transducers combined with dark-field or weakly focused optical illumination (Figure 12C). The use of high acoustic numerical aperture enables spatial resolution in the order of tens of micrometers, allowing detailed visualization of microvascular and structural features. These systems are typically mounted on articulated arms and acquire three-dimensional images through point-by-point raster scanning using a motorized stage. Owing to their high spatial resolution, mesoscopic and microscopic PAI are particularly suited for imaging superficial tissues and diagnosing skin disorders such as skin cancer and psoriasis.

## 9. Challenges, Design Principles, and Future Directions for Clinical Translation of Photoacoustic Imaging in Women’s Health

Despite significant progress, several challenges remain for the clinical translation of photoacoustic imaging (PAI) in women’s health. Limited optical penetration and signal attenuation in highly scattering tissues continue to restrict imaging performance in deep pelvic organs. In addition, variability in imaging systems, reconstruction algorithms, and data acquisition protocols limits reproducibility and cross-study comparability. In practical gynecological settings, imaging depth requirements vary depending on the application. For example, transvaginal or endocavity imaging typically requires penetration depths of ~1–3 cm, whereas transabdominal assessment of ovarian lesions may require depths exceeding 4–5 cm, where signal attenuation becomes more significant. In addition, variability in imaging systems, reconstruction algorithms, and data acquisition protocols limits reproducibility and cross-study comparability. Motion artifacts, heterogeneous tissue composition, and safety constraints associated with laser exposure further complicate clinical implementation, particularly in transvaginal, endoscopic, and intraoperative environments.

In addition to these general limitations, several challenges are uniquely relevant to pelvic and gynecological imaging. Optical access to deep pelvic organs is inherently restricted, often requiring transvaginal or endocavity approaches, which introduce constraints related to probe geometry, light delivery, and patient comfort. Quantitative interpretation of photoacoustic signals is further complicated by the heterogeneous composition of pelvic tissues and strong background absorption from blood-rich environments, which can reduce specificity. Physiological variability across the menstrual cycle also influences vascularity, oxygenation, and tissue optical properties, potentially affecting signal reproducibility and longitudinal assessment. Moreover, motion and pressure artifacts during endocavity imaging, along with requirements for probe sterility and disposability, pose additional clinical and engineering challenges. Finally, distinguishing between inflammation, fibrosis, hemorrhage, and malignancy remains difficult in cases where optical absorption spectra overlap, highlighting the need for advanced spectral unmixing and multiparametric imaging strategies.

Beyond imaging performance, practical implementation challenges must also be considered. Efficient laser delivery through compact probe geometries, particularly for transvaginal or endoscopic devices, remains a key technical barrier. Patient comfort and ergonomic constraints further limit probe size, scanning duration, and allowable energy exposure. In addition, sterilization requirements and compatibility with existing clinical workflows pose challenges for routine adoption, especially for intraoperative and office-based gynecological procedures.

To address these challenges, several key design principles should guide the development of next-generation PAI systems and contrast agents. First, contrast agents should exhibit strong and tunable optical absorption in the NIR/NIR-II window, enabling deeper tissue penetration and improved signal-to-background contrast [97]. Second, the development of biocompatible and biodegradable materials is essential to ensure safe clinical translation and minimize long-term accumulation [209]. Third, disease-specific targeting and activatable probe designs should be prioritized to enable biomarker-responsive signal generation, thereby improving diagnostic specificity beyond passive accumulation mechanisms [210]. Fourth, ratiometric and quantitative imaging strategies should be incorporated to reduce variability and enable reliable longitudinal monitoring [211].

From a materials perspective, recent innovations include hybrid nanostructures, organic/polymeric photothermal agents, and small-molecule activatable probes that offer improved specificity, stability, and functional imaging capabilities. Enzyme-responsive and microenvironment-sensitive probes (e.g., targeting MMPs, hypoxia, or inflammatory markers) represent a promising direction for achieving precision imaging of gynecological diseases such as endometriosis and ovarian cancer. These systems enable site-specific signal activation, addressing key limitations of conventional contrast agents.

On the instrumentation side, advances in miniaturized probe-based systems, including endoscopic and transvaginal PAI platforms, are critical for accessing deep-seated reproductive organs. These approaches are particularly promising for cervical imaging, intraoperative guidance during endometriosis surgery, and localized evaluation of pelvic disease. Integration with multimodal imaging techniques, such as ultrasound and fluorescence imaging, can provide complementary structural and functional information, enhancing diagnostic accuracy. Additionally, AI-assisted image reconstruction and analysis offer opportunities to improve signal sensitivity, reduce noise, and enable real-time clinical decision-making.

Ultimately, successful clinical translation of PAI will require coordinated progress in material design, system engineering, and standardization, including the development of robust imaging protocols, quantitative biomarkers, and regulatory frameworks. By addressing these challenges through targeted, application-specific innovations, PAI holds strong potential as a non-invasive, high-resolution imaging modality for early diagnosis, intraoperative guidance, and longitudinal monitoring in women’s health.

Recent advances in NIR-responsive 4D-printed shape-memory polymer composites highlight an emerging direction in which PAI can be integrated with deployable biomedical devices [212,213]. These systems enable real-time imaging during structural transformation and deployment, offering a unique opportunity for image-guided interventions in deep tissue environments. By incorporating photoacoustic-responsive components into smart biomaterials, it becomes possible to monitor device positioning, expansion, and functional performance non-invasively. Such approaches may be particularly relevant for minimally invasive gynecological applications, including implantable or expandable therapeutic platforms. The integration of PAI with advanced manufacturing technologies therefore represents a promising frontier for precision-guided diagnostics and therapy [214].

## 10. AI-Driven Photoacoustic Imaging

Although PAI demonstrated promising application in biomedical field, the trade-off between clinical application, image depth and resolution is still challenging. Recent advances in artificial intelligence (AI) have further enhanced photoacoustic image reconstruction and analysis, particularly for vascular segmentation and classification tasks [215]. Deep learning approaches, including convolutional neural networks, have demonstrated improved image quality and feature extraction, typically evaluated using metrics such as SSIM, PSNR, and Dice coefficient. Several recent studies have reported AI-assisted US/photoacoustic imaging frameworks for automated feature extraction and improved diagnostic performance [216,217]. However, domain shift between preclinical datasets and heterogeneous clinical data remains a significant challenge, limiting model generalizability. Addressing these issues will be essential for successful clinical translation. Recent studies have demonstrated the application of deep learning architectures such as U-Net, Res-U-Net, and GAN-based models for photoacoustic image reconstruction and processing, and representative recent studies are summarized in Table 7. These approaches also hold potential for improving lesion detection, segmentation, and characterization in gynecological imaging, particularly in complex pelvic environments where signal heterogeneity and overlapping contrast features are common [218,219].

**Table 7:** Applications of AI in photoacoustic image reconstruction and analysis.

Type	Year	AI Model Used	Key Contribution	Ref.
Image Analysis / Classification	2012	Pattern recognition algorithm (feature-based classification)	Classification of malignant vs benign ovarian tissue using co-registered PA + ultrasound imaging; demonstrated distinct absorption patterns and improved diagnostic differentiation	[220]
PAT Reconstruction	2018	CNN (U-Net-based)	First DL framework for sparse-data PAT reconstruction	[221]
PAT Reconstruction	2020	Res-U-Net	End-to-end inverse problem solving for PAT	[222]
PAT Reconstruction	2020	Hybrid CNN (Y-Net)	Combines raw + beamformed data for improved reconstruction	[223]
PAT Reconstruction	2020	CNN	Real-time reconstruction for microvascular imaging	[224]
PAM Reconstruction	2021	FD U-Net	Reconstruction from highly undersampled PAM data	[225]
Clinical Imaging Analysis	2021	Classical ML models (k-NN, SVM, Random Forest, Neural Network, Logistic Regression)	AI-based classification of rectosigmoid endometriosis using ultrasound-derived features demonstrated comparable performance to traditional statistical models in clinical dataset (n = 333)	[226]
Multimodal Image Analysis/Classification	2022	SVM	Combined PA/US features for classification of ovarian lesions; demonstrated improved diagnostic accuracy using multimodal feature extraction	[227]
Image Processing	2024	Noise2Noise CNN	Unsupervised denoising for PA images	[228]
Image Processing	2024	YOLOv8 + Pix2Pix	Artifact removal + segmentation in PA images	[229]
OC-PAM image analysis AI	2026	U-Net-type architecture	3D organoid segmentation	[230]

## 11. Conclusions

PAI has emerged as a powerful hybrid modality that bridges optical contrast with ultrasonic resolution, enabling non-invasive visualization of structural, functional, and molecular changes in biological tissues. Recent advances demonstrate the growing impact in women’s health applications, including cervical assessment, ovarian lesion characterization, pregnancy monitoring, and evaluation of reproductive tissue remodeling. By providing label-free sensitivity to hemoglobin, collagen, water content, and vascular architecture, PAI offers unique diagnostic information that complements conventional imaging techniques such as ultrasound and MRI. The incorporation of targeted molecular probes has further enhanced its diagnostic sensitivity and expanded its theranostic potential for gynecological diseases. Moreover, the integration of PAI with clinically compatible platforms, together with advances in spectral imaging, artificial intelligence-assisted analysis, and probe-based systems, has accelerated its transition toward translational and clinical use.

Despite remaining challenges related to imaging depth, system standardization, and regulatory approval, ongoing technological innovations and collaborative standardization efforts are steadily addressing these limitations. Future progress will likely be driven by multimodal imaging integration, quantitative imaging biomarkers, targeted contrast agents, and workflow-compatible clinical devices. Collectively, these advancements position PAI as a promising next-generation tool for early disease detection, personalized diagnosis, and image-guided management in gynecological and reproductive medicine, with the potential to significantly improve clinical outcomes in women’s health.

## Figures and Tables

**Table 1:** Comparison of major photoacoustic imaging modalities.

Modality	Imaging Principle	Spatial Resolution	Imaging Depth	Advantages	Limitations	Typical Applications
Optical-Resolution PAM (OR-PAM)	Optical focusing defines lateral resolution	~1–10 µm	~1–2 mm	Ultra-high resolution; capillary-level imaging	Limited depth due to optical scattering	Microvasculature, skin, superficial tissues
Acoustic-Resolution PAM (AR-PAM)	Acoustic focusing defines resolution	~30–100 µm	~3–5 mm	Improved depth vs OR-PAM; good vascular imaging	Lower resolution than OR-PAM	Subsurface vasculature, small animal imaging
Photoacoustic Computed Tomography (PACT)	Multi-element detection + reconstruction	~100–500 µm	~2–7 cm	Deep tissue imaging; whole-organ imaging	Lower resolution than PAM; reconstruction complexity	Tumor imaging, organ-level imaging, clinical translation
Endoscopic/Probe-based PAI (PAE/PAUS)	Integrated optical + acoustic probe	~50–300 µm	~1–3 cm (localized)	Minimally invasive; real-time imaging	Limited field of view; probe complexity	Gastrointestinal, gynecological, intraluminal imaging
Fabry–Perot/Optical Detection PAI	Optical interferometric ultrasound detection	High (depends on system)	Up to several cm	High sensitivity; wide-field imaging	Slower acquisition (scanning-based)	Preclinical imaging, vascular mapping

**Table 3:** Summary of representative preclinical and clinical photoacoustic imaging studies.

System/Contrast	Model	Imaging Type	Application	Key Outcome	Study Type	Ref.
OR/AR-PAM	Mouse	PAM	Microvascular imaging	Combined superficial + deep vessel visualization	Preclinical	[48]
Fabry–Perot PACT	Mouse (pregnancy)	PACT	Embryo imaging	Visualization of embryos and vasculature	Preclinical	[54]
Ring-array PACT	Mouse	PACT	Whole-body imaging	Organ-level vascular imaging	Preclinical	[55]
Balloon PAE probe	Animal model	PAE	GI imaging	Differentiation of normal vs diseased tissue	Preclinical	[63]
PAUS endoscopy	Rat	PAE	GI imaging	Real-time microvascular imaging	Preclinical	[64]
Endogenous Hb	Human palm	PAI	Vascular imaging	Depth-resolved vascular mapping	Clinical	[71]
Endogenous Hb	Animal brain	PAI	Functional imaging	Oxygenation mapping during stimulation	Preclinical	[83]
Endogenous Hb	Human breast	PACT	Breast imaging	3D vascular visualization	Clinical	[84]
SWNT–ICG	Mouse tumor	PAI	Tumor imaging	~300-fold sensitivity enhancement	Preclinical	[135]
Gold-coated CNTs	Mouse tumor	PAI/PTT	Theranostics	High contrast + photothermal ablation	Preclinical	[138]

Where GI is gastrointestine, PTT is photothermal therapy.

**Table 4:** A comparison of various imaging modalities with PAI for Women’s health.

Imaging Modality	Spatial Resolution	Imaging Depth	Contrast Agents	Primary Functional Parameters	Major Advantages	Main Limitations	Typical Gynecological Application
NIR-II Imaging	System-dependent (mm–cm scale)	Several mm to cm	Organic fluorophores, inorganic nanoparticles	Vascular morphology, hemodynamics	High spatial resolution, deep tissue penetration, real-time imaging	Contrast agent risks, high equipment cost	Tumor visualization, intraoperative imaging
PET	4–6 mm	Whole body	^18^F-FDG, ^18^F-NaF	Standardized uptake value, metabolic rate, perfusion	Whole-body imaging, high sensitivity, quantitative metabolic information	Radiation exposure, limited spatial resolution, high cost	Ovarian/cervical cancer staging and metastasis detection
MRI	0.3–1 mm	Whole body	Gadolinium-based agents	Degree of stenosis, hemodynamics	Non-invasive, radiation-free, high soft tissue contrast	Long scan time, contrast-related risks, limited microvascular detail	Uterine fibroids, adenomyosis, ovarian lesion characterization
CT	0.2–1 mm	Whole body	Iodinated contrast agents	Degree of stenosis, vessel wall characteristics	High spatial resolution, rapid 3D imaging	Ionizing radiation, nephrotoxicity risk, limited subtle pathology detection	Pelvic lesion localization, cancer staging
US	Tens of µm to several mm	Up to several cm	Typically, none (optional microbubbles)	Vascular morphology, blood flow	Real-time, radiation-free, low cost	Operator-dependent, resolution-depth tradeoff	First-line imaging for uterine, ovarian, and pregnancy assessment
PAI	Tens to hundreds of µm	Several mm to cm	Endogenous (Hb, melanin, lipid) and exogenous (dyes, nanoparticles)	Oxygen saturation, hemoglobin concentration, vascular morphology, metabolic rate	Combines optical contrast with ultrasound resolution, functional + structural imaging	Limited penetration, ongoing clinical standardization	Endometriosis detection, vascular remodeling, tumor angiogenesis, placental oxygenation, intraoperative guidance

**Table 5:** Representative PAI studies in gynecological applications and translational status.

Indication	Model	Modality	Comparator	Key Insight	Readiness	Refs.
Endometriosis	Animal	PACT/PAM	Laparoscopy, MRI	Enhanced lesion detection via vascular + molecular imaging	Preclinical	[16,20,112]
Uterine disorders	Animal/ex vivo	PACT	Ultrasound, MRI	Functional imaging of vascular remodeling and oxygenation	Early translational	[54,55]
Cervical cancer	Animal/pilot human	PACT	MRI, biopsy	Angiogenesis and hypoxia mapping improves tumor characterization	Early clinical	[83,84]
Ovarian lesions	Animal	PACT	MRI, ultrasound	Improved deep-tissue contrast with exogenous agents	Preclinical	[55,56]
Pregnancy/placenta	Animal/pilot human	PACT	Doppler ultrasound	Real-time placental oxygenation and perfusion monitoring	Translational	[54,83]

**Table 6:** Clinical Status of Major PAI Systems.

System	Clinical Status	Notes
MSOT Acuity/Acuity Echo (iThera Medical)	Clinical research/human trials	Used in clinical research centers for imaging inflammation, cancer, and vascular disease.
Imagio Breast Imaging System (Seno Medical)	FDA-cleared clinical device	Used clinically for breast lesion evaluation alongside ultrasound.
RSOM Explorer	Clinical research device	Used in human studies for dermatology and microvascular imaging.
Twente Photoacoustic Mammoscope	Clinical research prototype	Used in clinical breast cancer imaging trials.
Photoacoustic Endoscopy systems	Preclinical/early translational research	Still mainly experimental but progressing toward human trials.
Handheld PAI probes	Clinical research prototypes	Used in several pilot clinical studies but not yet standard devices.

## Data Availability

No new data were created or analyzed in this study.
